# Noninvasive Biomarkers for Assessing the Excitatory/Inhibitory Imbalance in Children with Epilepsy

**DOI:** 10.1523/JNEUROSCI.0520-25.2025

**Published:** 2025-09-26

**Authors:** Sakar Rijal, F. Kathryn King, Hmayag Partamian, Saeed Jahromi, M. Scott Perry, Crystal Cooper, Christos Papadelis

**Affiliations:** ^1^Neuroscience Research Center, Jane and John Justin Institute for Mind Health, Cook Children’s Health Care System, Fort Worth, Texas 76104; ^2^Department of Bioengineering, University of Texas at Arlington, Arlington, Texas 76010; ^3^Department of Pediatrics, Texas Christian University School of Medicine, Fort Worth, Texas 76104; ^4^Department of Psychiatry, UT Southwestern Medical Center, Dallas, Texas 75390; ^5^Department of Psychology, University of Texas at Arlington, Arlington, Texas 76010

**Keywords:** antiseizure medications, broadband gamma, electroencephalography, epilepsy, excitatory–inhibitory balance, magnetoencephalography, narrowband gamma, support vector machine

## Abstract

Epileptic seizures involve a cortical excitation–inhibition imbalance driven by dysfunctional interneurons that contribute to gamma oscillation generation. While impaired gamma oscillations are commonly reported in epilepsy, the dynamics of broadband and narrowband gamma as well as beta oscillations remain underexplored. These oscillations may serve as noninvasive electrophysiological markers associated with altered cortical dynamics, potentially reflecting underlying excitation–inhibition imbalance in epilepsy. Here, we recorded high-density electroencephalography and magnetoencephalography data to investigate visual stimuli-elicited cortical oscillations in 48 neurotypical (20 females) and 49 children (26 females) with epilepsy. We found that epilepsy is characterized by reduced amplitude and prolonged latency of evoked cortical response compared with controls after visual stimulation, with alterations in N1 peaks and M100, M150, and M250 components (*p* < 0.05). Additionally, source imaging revealed disrupted oscillatory features in epilepsy patients, including suppressed power, reduced amplitude, and increased latency in evoked and induced beta and gamma oscillations from the visual cortex (*p* < 0.05). These alterations were consistent across diverse epilepsy subtypes, including focal, generalized, and nonlesional epilepsy cases. Utilizing these differences, we developed a novel classification model that differentiates individuals with epilepsy from controls with high accuracy, offering potential clinical utility in epilepsy diagnosis. Our results suggest disrupted beta and gamma oscillations may be associated with impaired inhibitory mechanisms and altered cortical dynamics, potentially indicative of an excitation–inhibition imbalance. Our findings highlight the potential of noninvasive electrophysiological biomarkers to capture cortical dynamics possibly influenced by excitation–inhibition imbalance in epilepsy, supporting their use in early diagnosis and disease monitoring.

## Significance Statement

Diagnosing pediatric epilepsy is challenging as seizures often manifest subtly or mimic benign behaviors, complicating electroencephalogram interpretation. This underscores the need for noninvasive neurophysiological markers that can characterize cortical dysfunction. Excitation–inhibition imbalance is widely recognized as a key mechanism in the pathophysiology of epilepsy, with substantial evidence linking it to cortical gamma oscillations. Here, we identified consistent alterations in visually evoked and induced beta and gamma oscillations in children with epilepsy, which possibly reflect disturbances in inhibitory control and broader disruptions in cortical network dynamics. Features derived from these oscillations distinguished epilepsy from controls with good accuracy. These findings indicate that visual stimulus–related electrophysiological features are promising biomarkers for assisting early diagnosis and tracking disease progression in pediatric epilepsy.

## Introduction

The balance between excitatory and inhibitory (E/I) activity is fundamental to brain function, ensuring the stability of local and global network dynamics in the neurotypical brain (Duma et al., 2024). Conversely, disruption of E/I balance is a cardinal factor in the development of recurrent seizures (Gao et al., 2017; Xie et al., 2025). Yet, despite its recognized role, E/I imbalance is rarely integrated into epilepsy diagnostics. This gap is compounded by the challenge of diagnosing epilepsy, as up to 40% of first-time electroencephalograms (EEGs) in patients diagnosed later with epilepsy are initially interpreted as normal (Panayiotopoulos, 2005; Rowland and Lambert, 2022). Additionally, ∼30% of patients diagnosed with epilepsy who fail to respond to initial antiseizure medications (ASMs) are ultimately found not to have epilepsy (Jungilligens et al., 2021). Importantly, the diagnosis of epilepsy presents an even greater challenge in pediatric cohorts since seizures in young children often manifest subtly (Rose et al., 2019) or mimic benign behaviors (Stainman and Kossoff, 2020). This highlights the need for developing noninvasive biomarkers that provide a metric of cortical E/I imbalance, which enhances the early diagnosis of epilepsy, enabling timely therapeutic interventions.

E/I balance in epilepsy is currently assessed using positron emission tomography (PET) or proton magnetic resonance spectroscopy (^1^H-MRS), which provide indirect insights of E/I balance through receptor occupancy, neurotransmitter levels, and metabolism (Gonen et al., 2020; Steinbrenner et al., 2022). However, ^1^H-MRS and PET are challenging in children due to the need for sedation (McCarville, 2009) and concerns about radiation exposure (Blüml et al., 2022). In contrast, EEG and magnetoencephalography (MEG) provide noninvasive, child-friendly, high-temporal-resolution recordings of neuronal activity. Since these neuroimaging methods have complementary profiles of sensitivity (Chowdhury et al., 2015), their combined use in simultaneous recordings would enable a more comprehensive assessment of oscillatory dynamics, especially in children, where single-modality limitations may complicate accurate interpretation of sources (Corona et al., 2024).

Gamma oscillations (>30 Hz), recorded via EEG or MEG in response to sensory stimuli, are modulated by the interplay between excitatory and inhibitory neuronal activity and have been proposed as indirect functional markers of cortical E/I dynamics (Mujunen et al., 2022). These oscillations arise from the interplay of excitatory and inhibitory brain networks (Buzśaki and Wang, 2012) and are linked to cognitive functions (Manyukhina et al., 2021). Recent studies have distinguished two functionally distinct gamma band components: narrowband gamma (NBG) and broadband gamma (BBG), each offering complementary insights into E/I regulation. NBG is stimulus-driven and reflects rhythmic synchronization between excitatory pyramidal cells and inhibitory interneurons, serving as a functional marker of local inhibitory control (Perry et al., 2014). In contrast, BBG reflects broadband, nonoscillatory high-frequency activity associated with asynchronous spiking of local neuronal populations and is more indicative of disinhibition (Bartoli et al., 2019). Together, these signatures offer a nuanced view of cortical dynamics and can aid in detecting functional abnormalities in pediatric epilepsy when routine EEG is inconclusive. Similarly, beta oscillations (15–29 Hz) also contribute to E/I balance and interact with gamma rhythms to support inter-regional communication and hierarchical brain functions (Fuscà et al., 2023). While oscillatory measures do not directly quantify neurotransmitter concentrations or synaptic receptor activity, they are widely recognized as reliable noninvasive proxies of E/I balance, particularly in the context of epilepsy (Gaetz et al., 2011; Kujala et al., 2015; Ahmad et al., 2022).

Here, we noninvasively examined alterations in cortical rhythmic activity associated with pediatric epilepsy using simultaneous high-density EEG and MEG recordings during visual stimulation. We hypothesize that children with epilepsy will exhibit suppressed visual-evoked and induced cortical rhythmic activity, characterized by reduced power and amplitude as well as delayed cortical responses in visual-evoked fields (VEFs) and potentials after visual stimulation. Given the heterogeneity of pediatric epilepsy, including variations in ASM use and the presence or absence of structural lesions, we also examined how oscillatory alterations varied across focal, generalized, and nonlesional epilepsy subgroups to evaluate the robustness and generalizability of our findings.

## Materials and Methods

### Participants

We recruited 49 children and adolescents with a diagnosis of epilepsy (EP group) [age, 7−19 years; mean ± standard deviations (SD), 14.61 ± 2.9; 26 females] and 48 neurotypical healthy controls (control group; age, 10–18 years; mean ± SD, 13.58 ± 2.48; 20 females). Children from the EP group were recruited from the epilepsy clinic at Cook Children's Medical Center (CCMC). Neurotypical healthy controls were recruited from the local community. We assessed age differences between the two groups and found no differences (*p* > 0.05). In the EP group, 33 patients (67%) were diagnosed with focal epilepsy, while 16 patients (33%) had generalized epilepsy. Among the patients, 27 (55%) had either a normal magnetic resonance imaging (MRI) or findings unrelated to epilepsy, while 10 patients (20%) had an MRI-detected anatomical lesion related to epilepsy. MRI findings were unavailable for the remaining 12 patients (24%). The most common pathology among patients was malformation of cortical development, found in five patients (10%), followed by stroke in two patients (4%), tumor in one patient (2%), and hippocampal sclerosis in one patient (2%). One patient (2%) had both malformation of cortical development and gliosis. Pathology was unknown for 39 patients (80%), including those with a normal MRI and those without MRI data. For patients, epilepsy onset ranged from 6 months - 16 years (mean ± SD, 8.6 ± 4.49 years), whereas epilepsy duration ranged from 6 months to 18 years (mean ± SD, 6.38 ± 4.2 years). Epilepsy onset age was unknown for four patients. Patients received individualized ASM regimens with the number of concurrently prescribed ASMs ranging from one to five. This number reflects the total count of distinct agents per participant. Among the 49 patients, sodium channel blockers were the most common treatment mechanism, prescribed to 32 patients (65%). Additionally, 29 patients (59%) received at least one GABAergic (gamma-aminobutyric acid) ASM, while 14 patients (29%) were treated with at least one calcium channel blockers. The demographics of children with epilepsy are summarized in Table 1 and Extended Data Table S1.

**Table 1.** Patient demographics

Number	Sex (F/M)	Age (years)	Epilepsy onset (years)	Epilepsy duration (years)	Simultaneous (MEG/EEG)	MRI (yes/no)	Epilepsy type	Pathology	MRI findings	#ASMs
1	F	12	4	8	MEG only	Yes	Focal	NL	Normal	2
2	F	12	10	2	Yes	Yes	Focal	DEV	Tumor	4
3	F	14	9	5	Yes	Yes	Focal	DEV	FCD (P)	3
4	F	15	13	2	Yes	Yes	Focal	NL	Normal	4
5	M	16	2	14	Yes	Yes	Focal	NL	Normal	2
6	M	17	6	11	Yes	Yes	Focal	NL	Normal	3
7	M	18	6	12	MEG only	Yes	Focal	ACQ	Normal	1
8	F	13	1	12	Yes	Yes	Focal	DEV	FCD (F)	3
9	M	9	5	5	Yes	Yes	Generalized	DEV and ACQ	FCD and gliosis (F)	3
10	M	17	16	2	Yes	Yes	Focal	NL	Normal	3
11	F	13	Unknown	Unknown	Yes	Yes	Focal	NL	Normal	3
12	F	7	0.5	7	Yes	Yes	Focal	NL	Normal	1
13	M	16	9	6	Yes	Yes	Focal	ACQ	Stroke (F, P)	3
14	F	9	Unknown	Unknown	Yes	Yes	Generalized	NL	Normal	3
15	M	17	6	10	MEG only	Yes	Focal	NL	Normal	4
16	M	14	2	12	MEG only	Yes	Focal	NL	Normal	5
17	F	8	5	3	Yes	Yes	Focal	DEV	FCD (O)	3
18	M	17	11	7	Yes	Yes	Focal	ACQ	Sclerosis	3
19	M	12	7	6	Yes	Yes	Focal	DEV	FCD (P, O)	4
20	F	11	8	4	Yes	Yes	Focal	NL	Normal	4
21	M	17	0.5	18	MEG only	Yes	Focal	ACQ	Stroke (F)	2
22	F	10	4	7	Yes	Yes	Focal	DEV	FCD (F)	4
23	M	18	16	2	Yes	Yes	Focal	NL	Normal	3
24	M	17	12	6	Yes	No	Focal	Unknown	Unknown	2
25	M	14	12	3	MEG only	No	Generalized	Unknown	Unknown	1
26	F	16	12	5	Yes	Yes	Generalized	NL	Normal	1
27	F	17	13	5	Yes	No	Focal	Unknown	Unknown	1
28	F	15	6	9	Yes	Yes	Generalized	NL	Normal	2
29	F	12	6	8	Yes	Yes	Focal	NL	Normal	1
30	M	13	11	2	Yes	Yes	Generalized	NL	Normal	4
31	M	19	14	5	MEG only	Yes	Focal	NL	Normal	4
32	M	19	16	4	Yes	Yes	Focal	NL	Normal	1
33	M	14	6	8	Yes	Yes	Generalized	NL	Normal	2
34	F	19	Unknown	Unknown	MEG only	No	Generalized	Unknown	Unknown	1
35	F	16	15	1	Yes	No	Focal	Unknown	Unknown	1
36	M	16	13	5	Yes	Yes	Generalized	NL	Normal	3
37	F	18	6	14	MEG only	Yes	Generalized	NL	Normal	2
38	F	15	6	11	MEG only	No	Focal	Unknown	Unknown	1
39	F	19	Unknown	Unknown	Yes	Yes	Focal	NL	Normal	1
40	M	13	8	5	Yes	No	Focal	Unknown	Unknown	1
41	F	13	12	1	MEG only	Yes	Focal	NL	Normal	3
42	F	15	9	6	Yes	Yes	Focal	NL	Normal	3
43	M	18	12	6	MEG only	No	Generalized	Unknown	Unknown	1
44	F	15	1	15	Yes	No	Focal	Unknown	Unknown	1
45	M	14	13	1	MEG only	No	Generalized	Unknown	Unknown	1
46	F	16	10	7	Yes	No	Generalized	Unknown	Unknown	2
47	F	12	11	2	Yes	No	Generalized	Unknown	Unknown	4
48	F	15	13	3	Yes	Yes	Generalized	NL	Normal	1
49	M	14	13	0.5	Yes	Yes	Generalized	DEV	Normal	3

ACQ, acquired (i.e., gliosis, sclerosis, stroke); age, age at experimental procedure; ASMs, antiseizure medications; DEV, malformation of cortical development [i.e., focal cortical dysplasia (FCD), glioma, tumor]; EEG, electroencephalography; F, female; FL, frontal lobe; M, male; MEG, magnetoencephalography; MRI, magnetic resonance imaging; NL, nonlesional; O, occipital lobe; P, parietal lobe.

Participants and their legal guardians provided fully informed consent to participate in the study. The experimental procedures were approved by CCMC and North Texas Regional Institutional Review Boards.

### Experimental design

**Figure 1.**
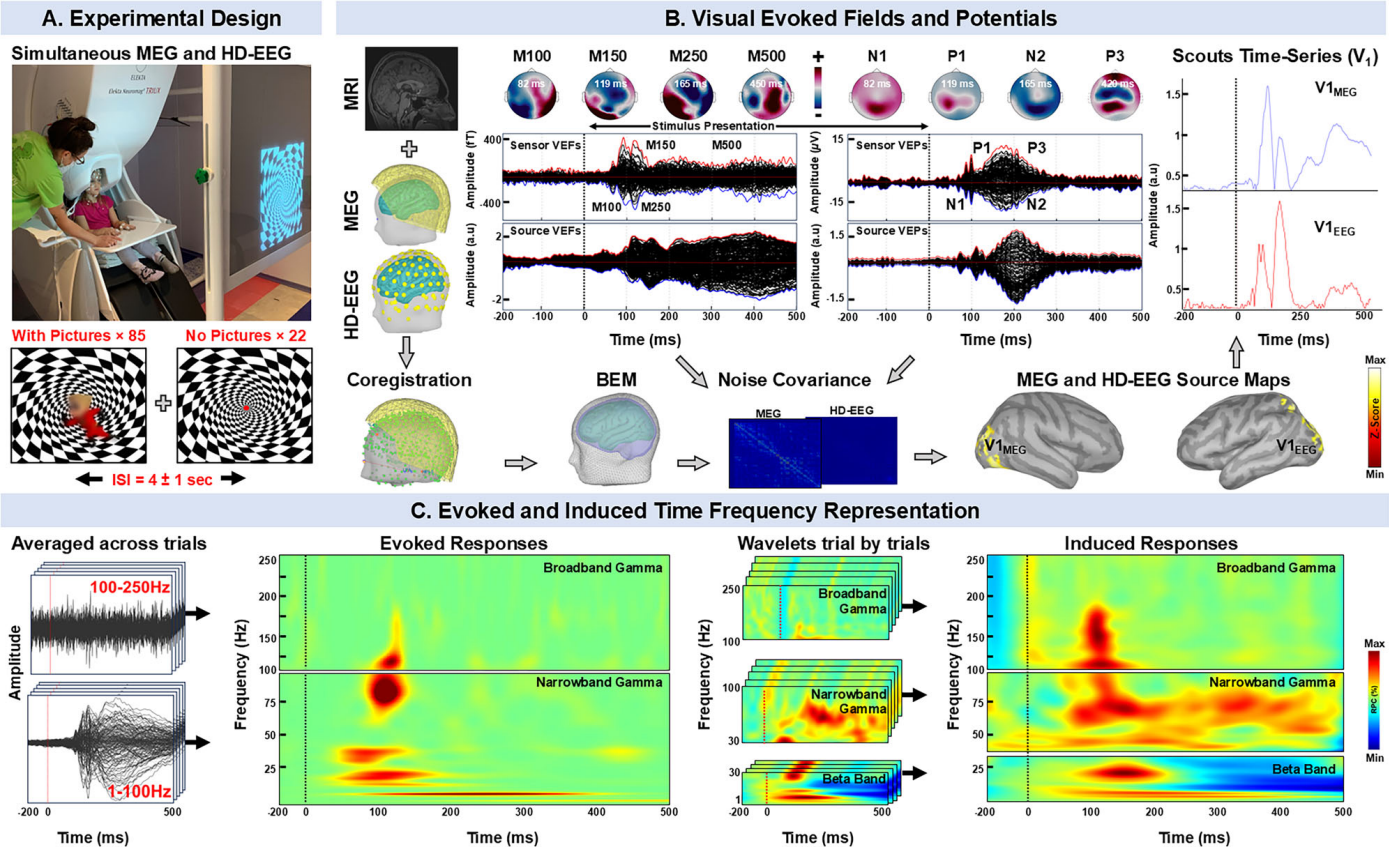
Data analysis pipeline. ***A***, Simultaneous recordings of high-density EEG (HD-EEG, 256 channels) and MEG (306 channels) were performed in a MSR; visual stimuli consisted of a checkerboard pattern with a diagram character (85 presentations; blurred to comply with copyright rules) at the center of the screen, along with pseudorandom 22 trials of the checkerboard pattern alone per EEG/MEG run with interstimulus interval of 4 ± 1 s. ***B***, Individual MRIs were used to create realistic head models using the boundary element method, which were coregistered with MEG and HD-EEG sensors. Sensor-level and source-level VEFs and VEPs were computed and highlighted components were utilized for further analysis (MEG, M100, M250, M250, M500; HD-EEG, N1, P1, N2, P3). Red and blue traces in the VEFs and VEPs represent the signal envelopes used to identify key components and peak responses. Noise covariance matrices were then computed from VEPs and VEFs separately for MEG and HD-EEG using prestimulus periods. Source maps were reconstructed, and virtual electrodes were implanted at the primary visual cortex (V_1_). ***C***, TFR of ESI and MSI at virtual channels was computed using complex Morlet wavelets across 1–100 Hz and 100–250 Hz. TFR maps were generated separately for evoked and induced responses. Evoked TFRs were derived from the average source maps across all trials, whereas induced TFRs were obtained by averaging wavelet transforms of source maps for individual trials. Induced activity was computed in three frequency bands (i.e., 1–30 Hz, 30–100 Hz, 100–250 Hz), with baseline correction applied from −0.2 to 0 s relative to stimulus onset. RPC, relative percentage change.

Simultaneous MEG and HD-EEG recordings were performed during visual stimuli presentations (Fig. 1*A*). MEG data were acquired using a 306-sensor (204 planar gradiometers and 102 magnetometers) whole-head system (TRIUX, Elekta Neuromag). Depending on the participant's head size, HD-EEG recordings were obtained using a Geodesic EEG System 400 (Magstim EGI) with either 256 electrodes (*n* = 77 participants) or 128 electrodes (*n* = 2 participants). All recordings were conducted at the MEG lab of CCMC. Recordings were performed in a dimly lit, one-layer magnetically shielded room (MSR) with a sampling rate of 1 kHz. Prior to recording, we placed five head position indicator (HPI) coils at known locations on the child’s head (i.e., one on each side of the forehead near the hairline; one on each mastoid bone; and one on the top of the head). The HPI coils were used to accurately determine the child’s head position relative to the MEG sensors. We used a motion tracking device (3SPACE FASTRAK, Polhemus) to digitize the fiducial landmarks, the participants' head shape (capturing over 500 surface points), and the locations of HPI coils. The locations of the positioning coils were recorded before the MEG experiment commenced. More details about the followed experimental procedures are provided previously (Corona et al., 2024).

For the HD-EEG recordings, we placed the Geodesic HD-EEG net on the child’s head and ensured that resistances were <10 kΩ for all electrodes. The coregistration of HD-EEG electrodes relative to the child’s head position was carried out by using the GeoScan Sensor Digitization System (Magstim EGI). During the recordings, participants were seated in a MEG-compatible chair and instructed to remain as still as possible. VEFs and visual-evoked potentials (VEPs) were recorded in response to visual stimuli presented on a screen placed in front of the participants (Fig. 1*A*). The setup included a MEG-compatible projector (Imedco) connected to a computer. Stimulus presentation was controlled by the Gentask module of the STIM package (NeuroScan). The visual stimuli included 85 age-appropriate captivating diagram characters (i.e., Disney, Minecraft, Airbender, SpongeBob, Mario, Phineas Ferb, Gumball, and Superhero) or faces overlaid on a checkerboard pattern, as well as 22 random trials of checkerboard patterns alone to minimize potential biases and ensure that the responses are not influenced by predictable patterns or sequences (Fig. 1*A*). To maintain consistency and control for variables known to affect VEFs/VEPs (i.e., contrast, angle, speed, and frequency of visual stimuli), these diagram characters were uniformly presented throughout the experiment for all participants. Although diagram stimuli may introduce some variability in visual responses, their inclusion was essential for maintaining engagement for our participants. To assess whether stimulus differences confounded group-level effects, we conducted separate analyses of both checkerboard-only trials and responses to individual diagram stimuli. For both stimulus conditions, fixation points or diagram characters were centered on the checkerboard to minimize variability from different gaze positions. Stimuli were presented for 1 s, followed by a variable interstimulus interval of 4 ± 1 s, in a pseudorandom order to prevent systematic effects that could arise from the order of presentation, such as anticipatory responses or habituation (Fig. 1*A*). Participants were instructed to focus on the fixation point or diagram characters to reduce artifacts from eye movements, blinks, and saccades. Foam wedges were placed between each participant's head and the inside of the dewar to provide comfort and to ensure stability and minimize any potential motion. If excessive movements were observed, the session was repeated. A member of the research team was always present inside the MSR during the recordings. The participants were monitored using a MEG-compatible video system to ensure compliance with instructions and to detect any notable artifacts, such as head movements, improper head positioning, or lapses in attention to the screen. The recording lasted ∼7 min per run; the total duration of the actual recording was ∼1 h (including breaks between runs). Electrocardiography (ECG) and electrooculography (EOG) data were simultaneously collected during MEG and HD-EEG recordings at a sampling rate of 1 kHz. ECG data were recorded using two leads placed on the sternum and the fifth intercostal space on the left side of the body, while EOG data were captured with two electrodes positioned above and below the eye on the right side of the face.

### MRI data acquisition

High-resolution, T1-weighted structural MRI sequences were acquired using a Siemens Skyra 3 T scanner with a 10-channel head coil. Specifically, a three-dimensional magnetization–prepared rapid acquisition gradient-echo sequence in the transverse plane was acquired with generalized autocalibrating partial parallel acquisition, with the following parameters: repetition time, 2,200 ms; echo time, 2.45 ms; flip angle, 8°; field of view, 250 mm; 176 slices; matrix size, 256 × 256; and acquired resolution, 1.0 × 1.0 × 1.0 mm. MRI data were unavailable for 12 patients and six neurotypical controls (Table 1, Table S1). For these individuals, age-matched MNI152 T1-weighted structural MRI templates were used for further analysis (Grabner et al., 2006).

### MEG and HD-EEG data analysis

We analyzed the MEG and HD-EEG data using *Brainstorm* (Tadel et al., 2011). MEG data were initially processed using temporal signal-space separation to suppress external noise and artifacts from nearby sources enhancing signal-to-noise ratio (Taulu and Simola, 2006). We then combined the MEG and HD-EEG data into a single .*fif* file by using an in-house *Python* code that spatially aligns the MEG and HD-EEG sensor locations and synchronize signals and triggers across the two acquisition systems (for details, see Corona et al., 2024). This integration was not performed to merge sensor data but to align and synchronize data streams for parallel analyses. Each modality was then processed independently to preserve its distinct spatial sensitivity and noise characteristics. MEG is primarily sensitive to sources with tangential orientation, while EEG captures sources with both tangential and radial orientation (Chowdhury et al., 2015; Chikara et al., 2023; Bastola et al., 2024). This complementary sensitivity is particularly important in pediatric populations, where higher anatomical variability and immature cortical folding can complicate single-modality source modeling (Ball et al., 2021). Hence, the use of simultaneous HD-EEG and MEG improves spatial resolution and increases the probability of capturing various ranges of source orientation and cortical depths. We further inspected the MEG and EEG data to remove bad channels; any time segments contaminated with movement or muscle tension artifacts were identified through visual inspection and excluded from further analysis. Additionally, biological artifacts, such as heartbeats and eyeblinks, identified through simultaneously recorded ECG and EOG data, were removed using the signal-space projection (SSP) method (Uusitalo and Ilmoniemi, 1997), which is well suited for correcting spatially consistent and time-locked biological artifacts, particularly in evoked paradigms (Atti et al., 2024). Both modalities underwent separate artifact correction procedures (e.g., EOG, ECG removal, channel-wise rejection) to preserve signal integrity before subsequent analysis. We selected SSP over ICA based on its computational efficiency, minimal reliance on assumptions, and its lower risk of removing task-related brain activity in time-locked analyses (Mutanen et al., 2016). Heartbeat and eyeblink artifacts were removed using SSP in a sequential, artifact-specific manner: first, cardiac artifacts were removed using heartbeat-only segments; next, blink artifacts were corrected using blink-isolated data, avoiding cross-contamination. This approach preserved neural signals while minimizing overcorrection. We removed the DC offset and applied a fourth-order bandpass infinite impulse response Butterworth filter with separate frequency ranges of 1–100 Hz and 100–250 Hz, as well as a 60 Hz notch filter and its harmonics (i.e., 120, 180, 240 Hz).

For the visual-evoked and induced cortical oscillations, we segmented each dataset into trials from −200 to 500 ms relative to the stimulus onset. Some MEG/EEG trials were lost due to technical difficulties and signal artifacts, leading to a reduction in the total number of analyzable trials (Table S1). We examined whether the number of artifact-free trials differed between the EP and control groups and found no differences (*p* > 0.05). These artifact-free trials from MEG and HD-EEG were then used for further analysis. Furthermore, to enhance signal focality and minimize the influence of volume conduction, skull thickness, and other noncortical contributions, HD-EEG trials were processed using common-average referencing (Muthukumaraswamy et al., 2010). In some participants (*n* = 22), stimulation-related artifacts were observed in the MEG and HD-EEG data. For those participants, we removed the time segment from −10 to 10 ms around stimulation onset and replaced those values with linear interpolations using MATLAB's *interp1* function; we excluded this period from further analysis to ensure homogeneity across all participants.

### Electric and magnetic source imaging

We performed electric and magnetic source imaging (ESI and MSI) on the averaged data of each participant using the dynamic statistical parameter mapping (dSPM), which is a noise-normalized solution based on the minimum norm estimate (Hämäläinen and Ilmoniemi, 1994) (Fig. 1*B*). We averaged the source imaging maps across runs for each participant to obtain an individualized average dSPM source model for each participant. We built realistic head models using boundary element model based on each participant's T1-weighted brain MRI scans or age-matched template MRIs to estimate the forward model (Fig. 1*B*). The models were generated with the *OpenMEEG* software and consisted of three surface layers (i.e., brain, inner skull, and outer skull). The dSPM solutions were estimated at the cortical surface (∼15,000 sources) reconstructed in *Brainstorm* using statistical parametric mapping (SPM) functions. The dSPM source solutions were computed separately for EEG and MEG using their respective noise covariance matrices and lead fields. This allowed us to retain the physiological specificity of each modality while using shared anatomical constraints for comparability across participants. The noise covariance was estimated on merged artifact-free prestimulus portions previously selected from the HD-EEG and MEG recordings. Grand-averaged cortical sources for each participant in the EP and control groups were calculated confirming that the maximum cortical sources of VEPs and VPFs were located within the primary visual cortex (V_1_). This was further validated by overlaying scouts from the left and right cuneus of the Desikan–Killiany atlas (Desikan et al., 2006) using *FreeSurfer* (Fischl, 2012). We averaged source maps for each condition and each participant and then projected each source map onto a common 5 mm grid based on the MNI brain (7,743 vertices) for group analysis. For further analysis, we focused on the maximum cortical activation of implanted scout in V_1_, calculating mean signals for each cortical vertices in V_1_ for each participant (An et al., 2018; Fig. 1*B*). We then compared the resulting source waveforms between the EP and control groups using an overlapping windowing approach. Specifically, we defined 25 ms windows (after stimulation onset) with 50% overlap, computed the mean amplitude for each window, and performed a “Wilcoxon rank-sum test” to assess group differences, followed by false discovery rate (FDR) correction for each window.

To analyze cortical components (determined from MEG) and peaks (determined from EEG) of cortical activity during visual stimulation in individuals with epilepsy and neurotypical controls, we first identified the upper and lower envelops of VEFs and VEPs at the sensor level from all the channels initially determined with MATLAB function *envelop.* Then, the maximum of upper envelopes and minimum of lower envelopes across all channels was determined and used to detect peaks and troughs. The MATLAB function *findpeaks* was applied in the detection of components or peaks in subject-specific grand–averaged VEFs and VEPs, with a minimum peak height constraint set to three standard deviations above the prestimulus amplitude (Song et al., 2024). The identified pairs of peaks and troughs were further evaluated visually. Additionally, we compared the peak amplitude and latency (relative to stimulus onset) of visual cortical components or peaks between individuals with epilepsy and controls in VEFs and VEPs. For VEFs, we analyzed the M100 (40–100 ms), M150 (101–150 ms), M250 (151–250 ms), and M500 (251–500 ms) components (Fig. 1*B*). For VEPs, in similar time range as VEFs, we examined the N1, P1, N2, and P3 peaks (Shigeto et al., 1998; Meeren et al., 2013). Furthermore, to investigate these components at the source level, we used virtual sensors from the left and right cuneus, defined in the Desikan–Killiany atlas, to reconstruct source VEFs and VEPs (Song et al., 2024). Same parameters were applied to analyze the peak amplitude and latency of the N1, P1, N2, P3 M100, M150, M250, and M500 components at both sensor and source levels (Fig. 1*B*).

### Time–frequency analysis

In both MEG and HD-EEG data, we determined the peak of the initial visual cortical response after stimulus onset for each participant individually. We generated virtual channels at the site of peak cortical activity (Fig. 1*B*) to enhance source-level accuracy by minimizing head movement artifacts (Velmurugan et al., 2022). These virtual sensors were modality-agnostic in their anatomical placement but derived independently from each participant's source-localized peak activity, as determined from EEG and MEG source models, respectively. Subsequently, we conducted time–frequency analysis of electric and magnetic brain activity at this virtual channel using complex Morlet wavelets to generate time–frequency representation (TFR) maps. To estimate evoked responses, we applied Morlet wavelets with five cycles per wavelet at center frequencies ranging from 1 to 100 Hz (in 1 Hz steps) to the averaged source waveforms (Fig. 1*C*). For the analysis of the BBG, we used Morlet wavelets with the same five-cycle width at center frequencies between 100 and 250 Hz in 1 Hz steps (Fig. 1*C*). We then corrected the TFR maps with respect to the baseline activity from −200 to 0 ms to estimate percentage change in power (i.e., relative power change). To estimate visual-induced fields (VIFs) and potential (VIPs) responses, we applied Morlet wavelets (using the same parameters as for evoked responses) to the source waveforms for individual trials from each participant (Fig. 1*C*). The estimation of TFRs for the induced response was performed using wavelet transforms at three frequency ranges (1–30, 30–100, and 100–250 Hz, separately) to minimize edge effects and 1/*f* power decay (Kujala et al., 2015). Furthermore, for counteracting 1/*f* power decay, we applied “spectral flattening” to the TFRs computation in the 1–30 Hz and 30–100 Hz ranges, where power in each frequency bin was multiplied by its corresponding frequency value to balance trade-off between power and frequency precision (Demanuele et al., 2007; Voytek et al., 2015). Without this compensation, low-frequency power could potentially obscure true neuronal responses in 1–100 Hz range (Miller et al., 2009). “Spectral flattening” was applied to the 1–30 Hz and 30–100 Hz frequency ranges and tends to overcompensate for frequencies beyond 100 Hz (Scheffer-Teixeira et al., 2013); thus, we left the 100–250 Hz range unadjusted. We then corrected the TFR maps for baseline activity in each trial across three frequency bands and averaged the baseline-corrected maps separately for each frequency range (Papadelis et al., 2023). Evoked and induced TFRs were averaged for each subject and then grand-averaged for all participants in the EP and control groups (Fig. 1*C*). Furthermore, to capture true neuronal oscillations in the different frequency bands for each evoked and induced TFRs, we defined oscillations as wavelets with peak amplitudes at least twice that of the background noise. By setting this threshold, we aim to increase the likelihood that the detected oscillations are genuine, minimizing the risk of false positives and improving the accuracy of our analysis (Inoue et al., 2004; Strigaro et al., 2021). Each TFRs for both evoked and induced responses were further evaluated visually by two reviewers (S.R. and F.K.K).

For each participant on subject-specific grand–averaged individual TFRs, we identified the peak frequency, latency (relative to the stimulus onset), and amplitude of the evoked and induced responses, separately for the beta, NBG, and BBG bands (Perry et al., 2014; Kujala et al., 2015). The peak frequency was identified as the frequency showing the largest power change after stimulus onset in each band (i.e., beta, NBG, and BBG); the peak latency was defined as the time–instance after stimulus onset showing the largest power change in each band; the peak amplitude was determined at the time–frequency bin corresponding to the peak frequency and time–instance in each band. Furthermore, we averaged the relative power change within the beta, NBG, and BBG to determine the average power change (in terms of magnitude) for each band; all these measures were used as “TFR features” of evoked and induced responses in subsequent statistical analyses (Fig. 1*C*). Furthermore, to address intraindividual variability in neuronal responses particularly in average power and peak amplitude, we applied min–max normalization across the participants (controls and EP group) using the formula *X*_normalized _= (*X* *−* *X*_min_)  / (*X*_max_ − *X*_min_), where *X* is the original feature value for a participant and *X*_min_ and *X*_max_ are the minimum and maximum value across participants, respectively (Duma et al., 2024). Additionally, for participants exposed to multiple diagram stimuli, we extracted key response features (i.e., peak frequency, latency, amplitude, and power) and compared them with those from the checkerboard-only stimulus. Trials with features deviating by more than three standard deviations from the checkerboard-only trials were excluded to minimize stimulus-related confounds.

### Statistical analysis

We performed statistical analysis of the visual-evoked and induced cortical oscillations data with *FieldTrip* toolbox in *Brainstorm* and MATLAB. For the time–frequency analysis, we analyzed the TFRs between subjects (i.e., EP vs control groups). All statistical tests were conducted on the poststimulus window (10–500 ms) using Monte Carlo permutation tests (1,000 permutations), with multiple comparisons corrected via cluster-based permutation testing (Meyer et al., 2021) in *FieldTrip*. Clusters were formed from neighboring pixels exceeding a *p* *<* *0.05* threshold. A reference distribution was generated with 1,000 permutations, and clusters were considered significant if they exceeded the 99th percentile of this distribution. For each participant, we extracted the power of relative responses within the time and frequency window where statistically significant clusters were observed and compared between EP and control groups (“Wilcoxon rank-sum test”). Additionally, we extracted the TFR features (i.e., peak frequency, peak latency, peak amplitude, and average power change of evoked and induced responses) for beta, NBG and BBG frequency bands and compared between the EP and control groups (“Wilcoxon rank-sum test”) followed by FDR correction (*p* < 0.05). To examine epilepsy subtype-specific effects, we performed subgroup analyses comparing children with focal and generalized epilepsy. TFR features of evoked and induced responses were compared across control, focal, and generalized epilepsy groups using pairwise Wilcoxon rank-sum tests with FDR correction (*p* < 0.05). Similarly, to address potential effects related to epilepsy pathology, we conducted a subgroup analysis comparing evoked and induced TFRs features between neurotypical controls and patients with nonlesional epilepsy (Table 1). Group differences were assessed using Wilcoxon rank-sum tests followed by FDR correction (*p* < 0.05). Successively, we performed “Spearman’s correlation” between the EP group TFR features of the beta, NBG, and BBG bands and clinical parameters (i.e., age of onset, epilepsy duration, and the number of ASMs), as well as age of participants during MEG/EEG experiment (correlations coefficient, *r*; *p* < 0.05). Furthermore, we performed multiple linear regression to assess the distinct contributions of each clinical parameter and patients' age to neurophysiological features (*p* < 0.05). Normalized feature values (accounting for interindividual variability) served as dependent variables, with age at epilepsy onset, patient age, the number of ASMs, epilepsy subtype (focal and generalized), and epilepsy duration as predictors (Duma et al., 2024). The model was implemented in MATLAB as follows: model = lm (feature matrix ∼ age of epilepsy onset + patient's age + number of ASMs + epilepsy duration + epilepsy subtype).

### Classification model

We implemented a supervised machine learning algorithm to classify participants into specific classes (i.e., epilepsy vs neurotypical controls). More specifically, we trained a support vector machine (SVM) classifier with a radial basis function kernel to distinguish participants based on their neurophysiological response patterns to visual stimulation. The performance of the classifier was evaluated using stratified 10-fold cross-validation, confusion matrix metrics, receiver operating characteristic (ROC) curve analysis, and significance testing by *χ*^2^ test (*p* < 0.05). To reduce overfitting, we applied a stratified 80/20 train–test split repeated over 1,000 iterations. In each iteration, models were trained on 80% of the data and tested on the remaining 20%, ensuring independence of the test set and unbiased estimates of model performance. We trained separate SVMs for three participant groups based on their recording modalities, using 80% of the data for training and the remaining 20% for testing: (1) children with both HD-EEG and MEG data available (Group 1), (2) children with only MEG data available (Group 2), and (3) children with only HD-EEG data available (Group 3). Each model used a regularization parameter of 10. For each group, we performed ANOVA on all normalized features that showed group-level differences between the EP group and neurotypical controls, including the peak amplitude and latency of VEP peaks (e.g., N1) and VEF components (e.g., M100, M150, M250). In addition, we analyzed normalized TFR features such as average power change, latency, and amplitude of both evoked and induced responses in the beta, NBG, and BBG bands. Furthermore, we retained only the features that showed statistically significant differences between classes before training the classifiers (Partamian et al., 2025). The ANOVA test provided *p* values for each feature, adjusted using the FDR correction. Significant features were identified based on these adjusted *p* values with importance scores calculated as the negative logarithm of the *p* value −log_10_(*p*). To mitigate potential bias from ASM use, we excluded features associated with ASMs in the EP group. The remaining significant features served as predictors, with class labels as the target. SVMs were validated using a stratified 10-fold cross-validation, ensuring balanced class representation in each fold and robust performance estimation across the dataset. To further strengthen reliability, this process was repeated across 1,000 random 80/20 stratified train–test splits, allowing us to assess the stability and generalizability of the model's performance across different data partitions. For each iteration, we defined true positive (TP) as correctly classified patients with epilepsy, true negative (TN) as correctly classified neurotypical controls, false positive (FP) as neurotypical controls incorrectly classified as patients with epilepsy, and false negative (FN) as epilepsy patients incorrectly classified as neurotypical controls. A confusion matrix was created for each group, and the model's performance was assessed using key metrics: (1) sensitivity = TP/(TP + FN); (2) specificity = TN / (TN + FP); (3) positive predictive value (PPV) = TP /  (TP + FP); (4) negative predictive value (NPV) = TN/(TN + FN); and (5) accuracy = (TP + TN) / (TP + TN + FP + FN). ROC curves were then generated to evaluate model's performance by calculating the area under the curve (AUC) for each group, with Youden's index identifying the optimal balance between TPs and FPs. Additionally, Chi-squared test (*χ*^2^) was utilized to assess the independence of predicted and actual classifications for all three groups.

Moreover, we used bootstrap-based method to statistically compare the AUCs for all three group models to determine whether the model performance was statistically different across the three groups (Pang et al., 2023). For this, we drew bootstrap sample (*n* = 10,000 times) from pool of AUC values for all three group models (1,000 iterations) and computed difference in AUC between all three models to build empirical difference distribution which was compared between groups for significance differences (*p* < 0.05). Moreover, to evaluate whether combining features from multiple modalities improves classification performance, we performed logistic regression on MEG and EEG from participants with simultaneous recordings (Group 1). First, we computed a base model using either EEG-only or MEG-only features from Group 1. Then, we built a full model that included both EEG and MEG features to evaluate whether the addition of the second modality significantly improved classification performance (Taneichi et al., 2011). Finally, a likelihood ratio test was performed to compare model deviances between MEG/EEG-only model and full model (*p* < 0.05) to evaluate whether integrating features across modalities provides additional predictive value compared with using a single modality alone.

## Results

We initially enrolled 105 participants, including 55 neurotypical healthy controls and 50 children with epilepsy. We excluded data from eight neurotypical controls due to high-frequency noise (>80 Hz) and one child with epilepsy who was later diagnosed with sunflower syndrome. Thus, we finally included data from 97 participants in data analysis. Among them, 78 had complete EEG and MEG recordings (EP group, 36; controls, 42). MEG data were available for 96 participants (EP group, 49; controls, 47), while due to technical difficulties, hardware issues, and participant compliance, HD-EEG recordings were obtained from 79 participants (EP group, 36; controls, 43). Refer to Table 1 and Table S1 for participant-specific MEG/HD-EEG recordings details.

To assess potential stimulus-driven variability originating from the inclusion of multiple diagram characters overlaid on checkerboard patterns, we assessed whether stimulus-driven variability influenced cortical responses. Most participants were presented with the same stimulus type (i.e., checkerboard with faces; *n* = 49), while additional diagram stimuli (e.g., SpongeBob, Airbender, Mario) were shown to overlapping subsets of 15–30 participants. A cluster-based permutation test revealed no differences in VEPs or VEFs across stimulus types (*p* > 0.05), indicating that variability in visual stimuli did not show group-level heterogeneous cortical responses. To further validate this, we compared key features of evoked and induced responses peak frequency, latency, amplitude, and power across diagram stimuli within both groups and observed no differences (*p* > 0.05, Wilcoxon rank-sum; Fig. S2*B*,*C*). Additionally, for participants subjected to multiple stimuli, we compared each cartoon's response features with those from the checkerboard-face condition, excluding runs with deviations exceeding three standard deviations. Only runs with stable response features were retained for sensor- and source-level analyses of VEFs, VEPs, and evoked and induced responses. This approach ensured that the observed group-level effects reflect underlying neurophysiological differences rather than variability in visual stimulus properties.

### Temporal and amplitude variations in visual-evoked responses

**Figure 2.**
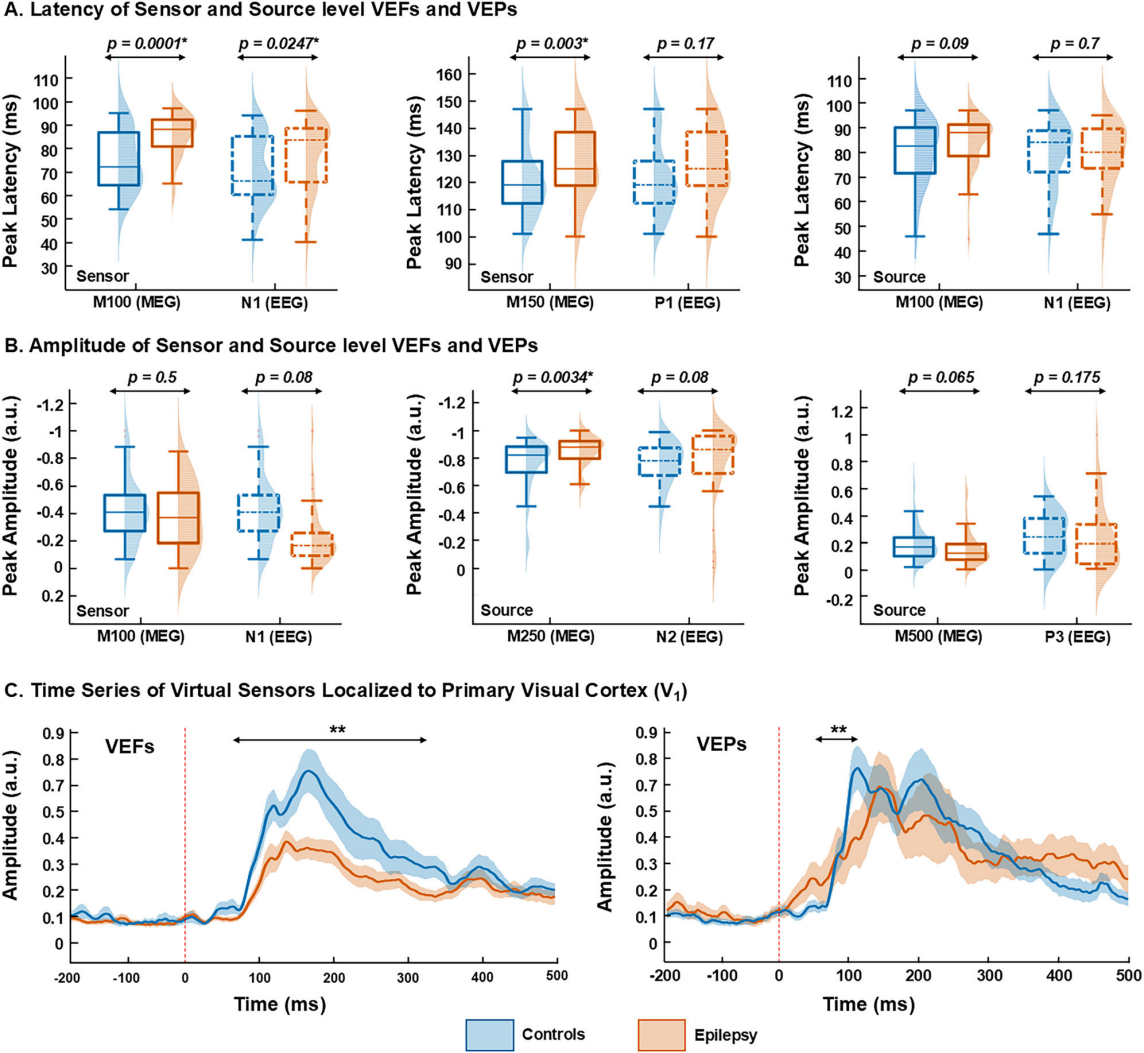
Comparison of VEFs and potentials. ***A***, Box plots compare peak latency between neurotypical controls (blue) and epilepsy patients (orange) for M100 and M150 and N1 and P1 in sensor and source VEFs and VEPs. ***B***, Box plots compare peak amplitude between controls and epilepsy patients for M100 and M250 and N1, P1, and P3 in sensor and source VEFs and VEPs. ***C***, Virtual sensors time series (filtered 1–100 Hz) localized to the primary visual cortex (V_1_) is displayed separately for VEFs and VEPs in the controls (blue trace) and epilepsy (orange traces) (VEFs, controls, *n* = 47; epilepsy, *n* = 49; VEPs, controls, *n* = 43; epilepsy, *n* = 36). Boxplots display the median (horizontal line) and interquartile range (25th and 75th percentiles as box edges), and whiskers extend to the minimum and maximum values, excluding outliers. Outliers are indicated as individual points beyond 1.5 times the interquartile range. *p < 0.05, “Wilcoxon’s rank-sum test” and FDR correction.

To ensure valid group-level comparisons, only VEFs and VEPs runs unaffected by stimulus variability were included in the final analyses (Fig. S2). “Wilcoxon rank-sum tests” showed significant differences in either amplitude or latency of VEF components and VEP peaks between the EP group and controls (Fig. 2).

At the sensor level, VEFs and VEPs revealed specific latency differences between groups. In VEFs at the sensor level (sensor VEFs), the M100 component showed a longer latency in the EP (compared with the control) group (M100, 88 ± 9 ms vs 72 ± 12 ms; *p* = 0.0001, FDR-corrected; Fig. 2*A*). Similarly, for VEPs at the sensor level (sensor VEPs), the N1 peak showed a longer latency in the EP (compared with the control) group (N1, 84 ± 15 ms vs 66 ± 14 ms; *p* = 0.024, FDR-corrected; Fig. 2*A*). The M150 component of sensor VEFs also showed a latency delay in the EP group compared with controls (125 ± 12.8 ms vs 119 ± 12.9 ms; *p* = 0.003, FDR-corrected; Fig. 2*A*). No such latency delay was observed for the P1 peak for the sensor VEPs between EP and controls (P1, 120 ± 14 ms vs 115 ± 12 ms; *p* = 0.17; Fig. 2*A*). Furthermore, analyses of sensor- and source-level VEFs and VEPs revealed no group differences in the latency of cortical components M250 and M500, as well as N2 and P3 peaks (all *p* > 0.05).

For VEFs, at the source level (source VEFs), the M250 component showed reduced peak amplitude in the EP group compared with controls (M250, −0.87 ± 0.19 a.u. vs −0.81 ± 0.16 a.u.; *p* = 0.003, FDR-corrected; Fig. 2*B*). No group difference was observed in the N2 peak amplitude of the source VEPs between EP and controls (N2, −0.86 ± 0.27 a.u. vs −0.77 ± 0.15 a.u.; *p* = 0.08; Fig. 2*B*). No difference was found in the amplitude of the M500 component of source VEFs between the EP group and controls (0.12 ± 0.13 a.u. vs 0.17 ± 0.16 a.u.; *p* = 0.06; Fig. 2*B*). Similarly, P3 peak amplitude of source VEPs did not differ between groups (0.18 ± 0.24 a.u. vs 0.23 ± 0.15 a.u.; *p* = 0.17; Fig. 2*B*). Additionally, no group differences were found in the amplitudes of M100 and M150 components as well as N1 and P1 peaks (all *p* > 0.05).

Grand-average VEF and VEP waveforms from virtual sensors in V_1_ are presented for both groups (Fig. 2*C*). Amplitude differences were assessed using 25 ms overlapping windows (50% overlap), “Wilcoxon rank-sum” test, and “FDR” correction (see Materials and Methods). We observed greater amplitude in the visual fields' component for controls (compared with EP) between 50 and 350 ms poststimulus (*p* < 0.05, “FDR-corrected”; Fig. 2*C*). Similarly, the EP group showed reduced amplitude in VEPs compared with controls 50–125 ms poststimulus (*p* < 0.05, “FDR-corrected”; Fig. 2*C*). Grand-averaged VEFs and VEPs are presented for both sensor and source space in a separate analysis of the checkerboard-only condition (Fig. S1*B*,*C*), which showed well-established spatiotemporal patterns across groups and thus supporting the consistency of evoked response independent of overlaid diagram stimuli.

### Evoked responses TFRs analysis in V_1_

**Figure 3.**
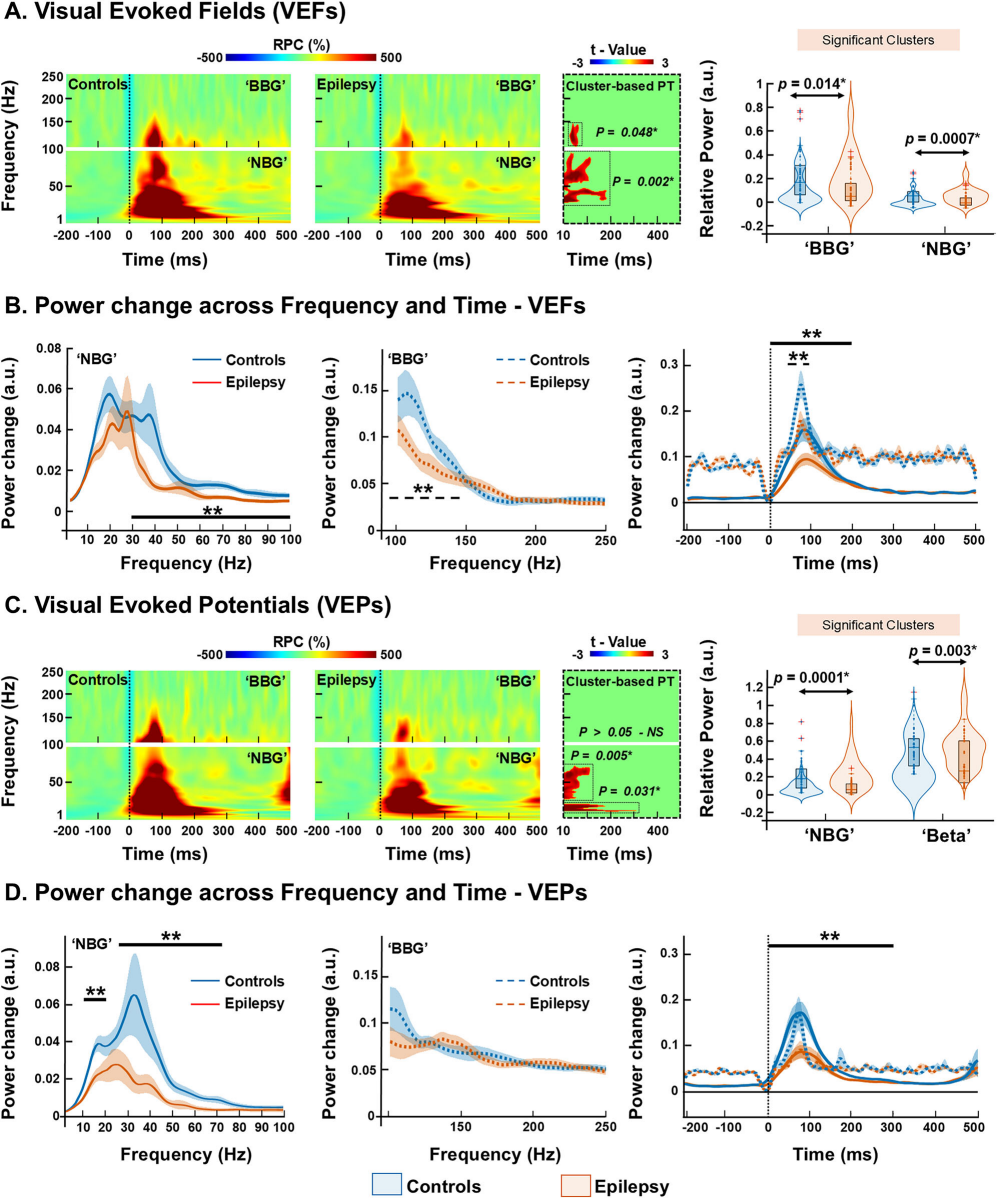
Grand-averaged TFR of VEFs and potentials. ***A***, Group average TFRs derived from VEFs shown separately for BBG (100–250 Hz, top panel) and NBG (1–100 Hz, bottom panel) for neurotypical controls (left panel, *n* = 47) and epilepsy (right panel, *n* = 49). The color scale represents the percentage of power change relative to the baseline (−200 to 0 ms), with global minimum and maximum used for scaling. The black dotted line at 0 ms marks stimulus onset, and blue traces near stimulus onset indicate interpolated values to account for stimulus-related artifacts. Monte Carlo permutation tests (1,000 permutations), corrected for multiple comparisons using cluster-based correction (*p* < 0.05). The black dotted box highlights the TFRs with statistically significant differences, corresponding to the *t* value distribution map. Significant differences were tested between 10 and 500 ms. Violin plots overlaid on box plots comparing the relative power change (RPC) between controls (blue) and epilepsy patients (orange). Normalized RPC values were extracted for each subject within the time-windows and frequency bands that showed statistically significant differences across all participants. ***B***, Group-normalized average power for controls and epilepsy averaged from the 10–190 ms poststimulus time-window separately for NBG (left panel) and 40–90 ms poststimulus time-window for BBG (middle panel). Group-normalized average power time course for NBG and BBG ranges (right panel; line represents groups; shading reflects SEM). ***C***, VEPs, shown separately for BBG (top panel) and NBG (bottom panel) for controls (left panel, *n* = 43) and epilepsy (right panel, *n* = 36). Monte Carlo permutation tests (1,000 permutations), corrected for multiple comparisons using cluster-based correction (*p* < 0.05). The black dotted box highlights the TFRs with statistically significant differences, corresponding to the *t* value distribution map. Violin plots overlaid on box plots comparing the normalized RPC between controls and epilepsy patients. Normalized RPC values were extracted for each subject within the time-windows and frequency bands that showed statistically significant differences across all participants. ***D***, Group-normalized average power for controls and epilepsy averaged from the 10–120 ms poststimulus time-window separately for NBG (left panel) and 10–500 ms poststimulus time-window BBG (middle panel). Group-normalized average power time course for NBG and BBG ranges (right panel; line represents groups; shading reflects SEM). Boxplots display the median (horizontal line), interquartile range (25th and 75th percentiles as box edges), and whiskers extend to the minimum and maximum values, excluding outliers. Outliers are indicated as individual points beyond 1.5 times the interquartile range. The overlaid violin plots show the distribution density of the data for each group. *p*, *p* value; *p < 0.05, “Wilcoxon’s rank-sum test”; SEM, standard error of the mean.

Visual stimulation resulted in a prominent cortical response at the virtual sensor located within the V_1_ for all participants (Fig. 1*B*). A cluster-based permutation test was applied after selecting the a priori poststimulus time-window (10–500 ms) and frequency bands (1–100 Hz “NBG” and 100–250 Hz “BBG,” separately). In VEF-derived evoked TFRs, we observed suppressed relative power in the EP group (compared with controls), particularly in the NBG and BBG (Fig. 3*A*). This power suppression was seen between ∼10 and ∼190 ms poststimulation, in the ∼30 and ∼95 Hz range for NBG (*p* = 0.002, corrected), and later between ∼40 and ∼90 ms, within the ∼100 and ∼150 Hz range for BBG (*p* = 0.04, corrected; Fig. 3*A*). Group-normalized relative change in power within this time and frequency window for NBG was 0.09 ± 0.16 a.u. in controls vs 0.041 ± 0.08 a.u. in the EP group (*p* = 0.0007), and for the BBG, it was 0.19 ± 0.21 a.u. in controls versus 0.07 ± 0.14 a.u. in the EP group (*p* = 0.014; Fig. 3*A*). To assess the impact of visual stimulation on power changes between groups, we extracted and averaged power values within time-windows showing group differences. Specifically, we quantified NBG responses within 10–190 ms poststimulus onset and broadband gamma BBG activity within the 40–90 ms window (Fig. 3*B*). Additionally, to enable a precise characterization of the temporal evolution of power changes in each group, we averaged power values within defined time bins (−200 to 500 ms) and projected the normalized power changes over time. This allowed us to understand precise depiction of how visual-evoked responses differ across groups in both frequency and time-resolved dimensions (Fig. 3*B*). Suppression of relative power in the EP group was also evident in response to the checkerboard-only trials, specifically within the NBG range (1–100 Hz) for VEF-derived evoked TFRs. This suppression of power was observed between ∼119 and ∼215 ms poststimulation, within the ∼35 and ∼60 Hz range for NBG (*p* = 0.037, corrected; Fig. S1*D*).

Similarly, analysis of evoked TFRs determined from VEPs showed suppressed relative power in the gamma band in the EP group (compared with controls; Fig. 3*C*). Cluster-based permutation test on the a priori selected poststimulus time-window (10–500 ms) and frequency bands (1–100 Hz and 100–250 Hz, separately) revealed reduced relative power in the NBG and beta bands for the EP group (compared with controls; Fig. 3*C*). The suppression in NBG (∼30 and ∼70 Hz; *p* = 0.005, corrected) was observed between ∼10 and ∼120 ms poststimulus (Fig. 3*C*). In this time and frequency for NBG, relative power was 0.12 ± 0.17 a.u. in controls versus 0.03 ± 0.06 a.u. in EP patients (*p* = 0.0001). Similarly, the suppression in beta band (∼15 and ∼20 Hz; *p* = 0.031, corrected) was observed between ∼10 and ∼300 ms poststimulus (Fig. 3*C*). In the beta band, relative power was 0.41 ± 0.2 a.u. in controls versus 0.17 ± 0.22 a.u. in EP patients (*p* = 0.003). No such significant cluster was observed for the BBG (*p* > 0.05; Fig. 3*C*). To assess the impact of visual stimulation on power changes between the control and EP groups, we extracted and averaged power values within time-windows that showed statistically significant differences. Specifically, NBG and beta band responses were quantified within the 10–120 ms poststimulus window, where overlapping group differences for NBG and beta bands were evident (Fig. 3*D*). In contrast, BBG activity was averaged across the entire 10–500 ms window, as no differences were observed between groups (Fig. 3*D*). Additionally, to characterize the temporal evolution of power changes in each group, we averaged power values within defined time bins (−200 to 500 ms) and projected the normalized power changes over time. This allowed us to precisely depict how visual-evoked responses differ between groups in both frequency- and time-resolved dimensions (Fig. 3*D*). Additionally, suppression of relative power in the EP group was also evident in response to the checkerboard-only trials, specifically within the NBG range (1–100 Hz) for VEP-derived evoked TFRs. This suppression of power was observed between ∼10 and ∼90 ms poststimulation, within the ∼30 and ∼50 Hz range for NBG (*p* = 0.01, corrected; Fig. S1*E*).

### Characteristics of evoked responses between EPs and controls

A cluster-based permutation test of the evoked TFRs revealed the most significant group differences in power between ∼10 and ∼200 ms following stimulus onset for both VEFs and VEPs (Fig. 3). Within this window, TFR features (average power change, peak frequency, amplitude, and latency) were extracted and compared between groups (“Wilcoxon rank-sum test” followed by FDR correction; *p* < 0.05; Kujala et al., 2015; Papadelis et al., 2023). Furthermore, these TFR features were separately correlated with age in both EP and controls and with clinical parameters in the EP group (Spearman's correlation, *p* < 0.05).

**Figure 4.**
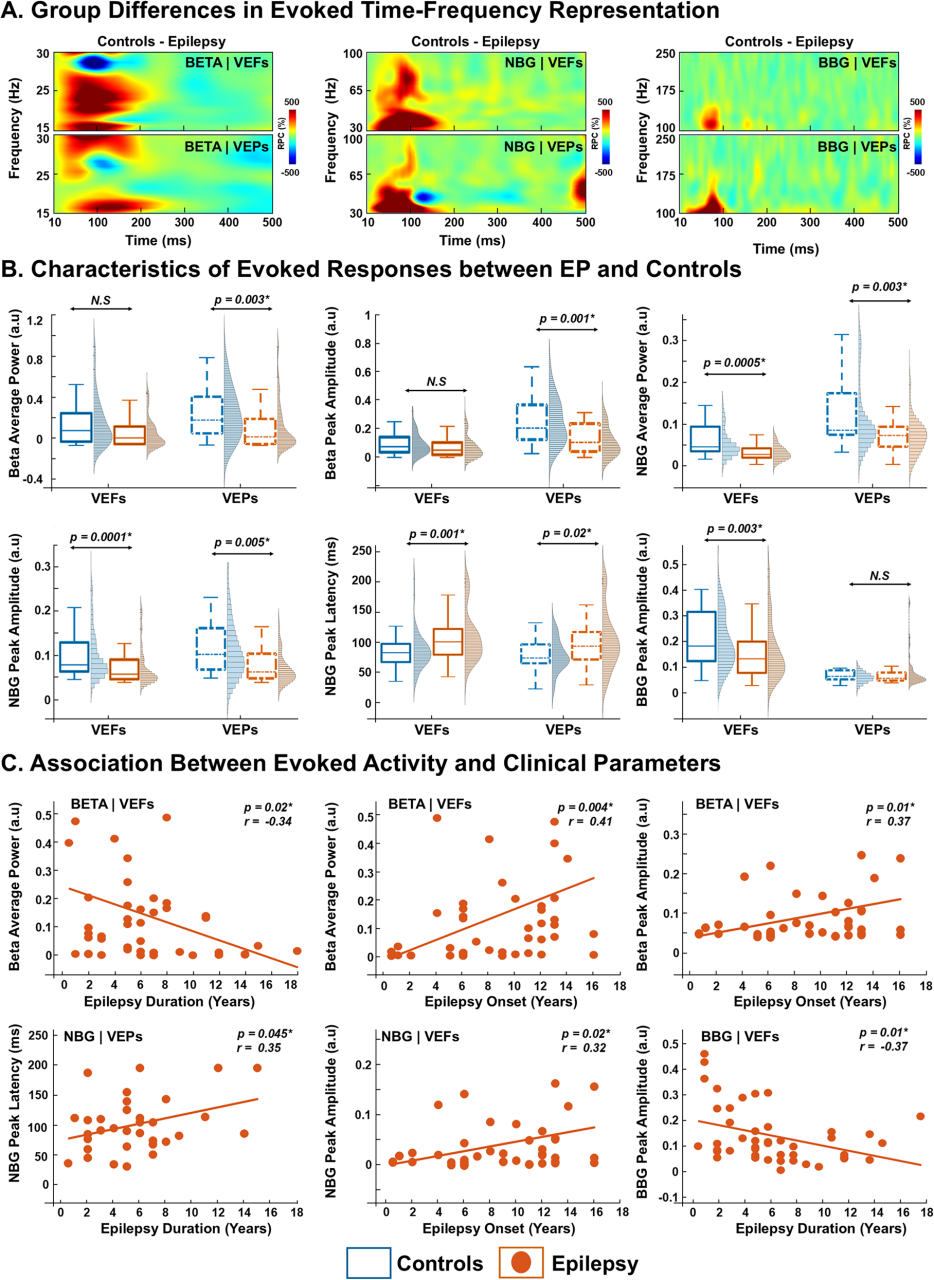
Features of evoked beta, narrowband, and broadband oscillations in the healthy controls and epilepsy patients determined by VEFs and potentials. ***A***, Time–frequency plot show difference in grand-averaged visual responses between neurotypical controls and epilepsy for beta, narrowband gamma (NBG), and broadband gamma (BBG) responses determined separately form VEFs (top panel), and VEPs (bottom panel). ***B***, Comparison of evoked features (average power, peak amplitude, and peak latency) of beta, NBG, and BBG responses between controls (blue) and epilepsy (orange) determined with MEG (VEFs, solid traces) and EEG (VEPs, dotted traces). ***C***, Scatterplots show the relationship between evoked features (determined from VEFs or VEPs) in different frequency ranges (beta, NBG, and BBG) and clinical variables (i.e., age of epilepsy onset and duration), for epilepsy patients (orange dots). In the figure, boxplots display the median (horizontal line) and interquartile range (25th and 75th percentiles as box edges), and whiskers extend to the minimum and maximum values, excluding outliers. Outliers are indicated as individual points beyond 1.5 times the interquartile range. *p*, *p* value; *p < 0.05, Wilcoxon's rank-sum test followed by FDR correction. *r*, Spearman’s correlation coefficient. The color scale represents percentage power change relative to the baseline (−200 to 0 ms).

To investigate group differences in evoked activity, we first computed the difference in poststimulation responses between groups (Fig. 4*A*). Grand-averaged TFRs in beta band, NBG, and BBG ranges were subtracted between controls and EP for both VEFs and VEPs revealing distinct patterns of group-level differences following visual stimulation (Fig. 4*A*). In the beta band, group-normalized average power in the VEPs was higher in controls compared with EPs (0.25 ± 0.23 vs 0.08 ± 0.22 a.u.; *p* = 0.003, FDR-corrected; Fig. 4*B*). Similarly, for VEPs normalized peak amplitude was greater in controls than in EPs (0.20 ± 0.18 vs 0.105 ± 0.21 a.u.; *p* = 0.001, FDR-corrected; Fig. 4*B*). No group differences were observed in peak frequency or peak latency (Fig. S3*A*; *p* > 0.05). In the EP group, a moderate negative correlation was observed between average beta power derived from VEFs and epilepsy duration (*r* = −0.34; *p* = 0.02; Fig. 4*C*). Conversely, average beta power (*r* = 0.41; *p* = 0.004) and peak amplitude (*r* = 0.37; *p* = 0.01) showed moderate positive correlations with age at epilepsy onset (Fig. 4*C*). Similarly, negative correlation was observed between beta peak frequency and ASM count (VEPs, *r* = −0.34; *p* = 0.037; Fig. S3*A*).

For NBG, the control participants exhibited a greater increase in group-normalized NBG power compared with the EP group, for both VEFs and VEPs (VEFs, 0.04 ± 0.15 a.u. vs 0.02 ± 0.05 a.u.; *p* = 0.0005; VEPs, 0.08 ± 0.23 a.u. vs 0.07 ± 0.11 a.u.; *p* = 0.003, FDR-corrected; Fig. 4*B*). Additionally, the group-normalized peak amplitude of NBG was higher in controls compared with the EP for both VEFs and VEPs (VEFs, 0.03 ± 0.14 a.u. vs 0.01 ± 0.06 a.u.; *p* = 0.0001; VEPs, 0.06 ± 0.2 a.u. vs 0.02 ± 0.08 a.u.; *p* = 0.005; FDR-corrected; Fig. 4*B*). NBG peak latency relative to stimulus onset was shorter in controls than in EPs, as measured from both VEFs (83 ± 32 vs 102 ± 43 ms; *p *= 0.001) and VEPs (74 ± 26 vs 95 ± 50 ms; *p *= 0.02; Fig. 4*B*). No group differences were observed for NBG peak frequency determined from VEFs and VEPs (Fig. S3*B*; *p* > 0.05). Similarly, NBG peak latency and amplitude exhibited moderate positive correlations with clinical variables. Peak latency was positively correlated with epilepsy duration (VEPs, *r* = 0.35; *p* = 0.045), while peak amplitude was positively correlated with age at epilepsy onset (VEFs, *r* = 0.32; *p* = 0.02; Fig. 4*C*).

For the BBG, controls exhibited a higher peak amplitude compared with the EPs determined from VEFs (VEFs, 0.16 ± 0.2 a.u. vs 0.11 ± 0.1 a.u.; *p* = 0.003, FDR-corrected; Fig. 4*B*). No differences were observed between groups in power, peak frequency, and peak latency as determined by both VEFs and VEPs (Fig. S3*C*). Additionally, moderate negative correlation was observed between the BBG peak amplitude, as determined from the VEFs, and the epilepsy duration in the EP group (*r* = −0.37; *p* = 0.01; Fig. 4*C*).

### Induced responses TFR analysis in V_1_

Group-averaged induced TFRs were derived from subject-specific average trials, aligned to the same virtual channel location as the evoked response in V_1_ (see Materials and Methods). Induced TFRs were analyzed separately for beta band, NBG, and BBG groups across predefined time (10–500 ms) and frequency windows, followed by cluster-based permutation analysis (*p* < 0.05).

**Figure 5.**
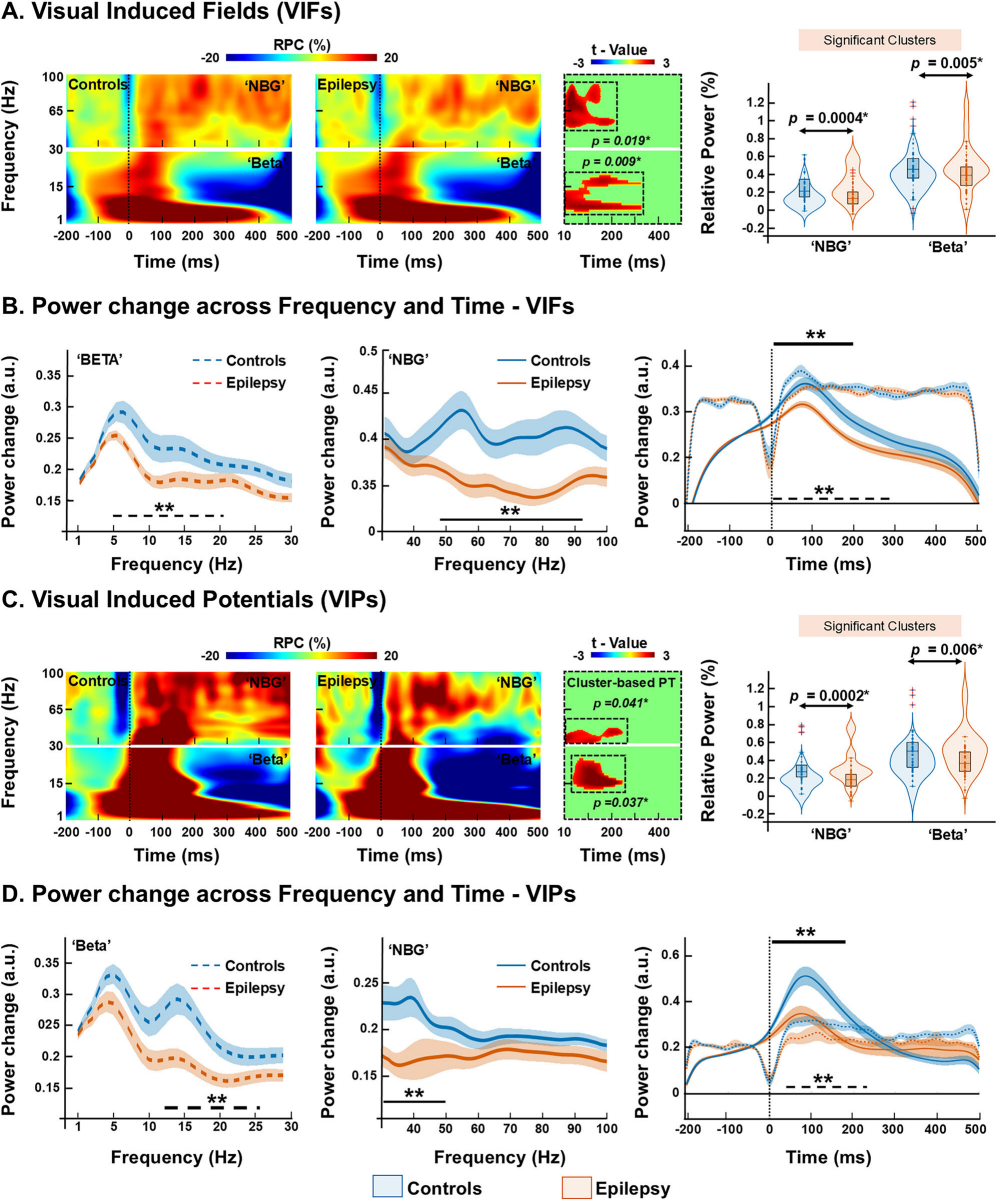
Grand-averaged TFR of VIFs and potentials. ***A***, Group average TFRs derived from VIFs shown separately for NBG (30–100 Hz, top panel) and beta band (1–30 Hz, bottom panel) for neurotypical controls (left panel, *n* = 47) and epilepsy (right panel, *n* = 49). The color scale represents percentage power change relative to baseline (−200 to 0 ms), with global minimum and maximum used for scaling. The black dotted line at 0 ms marks stimulus onset and blue traces near stimulus onset indicate interpolated values to account for stimulus-related artifacts. Monte Carlo permutation tests (1,000 permutations), corrected for multiple comparisons using cluster-based correction (*p* < 0.05). The black dotted box highlights the TFRs with statistically significant differences, corresponding to the *t* value distribution map. Significant differences are tested between 10 and 500 ms. Violin plots overlaid on box plots comparing the relative power change (RPC) between controls (blue) and epilepsy patients (orange). Normalized RPC values were extracted for each subject within the time-windows and frequency bands that showed statistically significant differences across all participants. ***B***, Group-normalized average power for controls and epilepsy averaged from the 50–300 ms poststimulus time-window separately for beta (left panel) and 100–200 ms poststimulus time-window for NBG (middle panel). Group-normalized average power time course for beta and NBG ranges (right panel; the line represents groups; shading reflects SEM). ***C***, VIPs, shown separately for NBG (top panel) and beta band (1–30 Hz, bottom panel) for controls (left panel, n = 43) and epilepsy (right panel, *n* = 36). Monte Carlo permutation tests (1,000 permutations), corrected for multiple comparisons using cluster-based correction (*p* < 0.05). The black dotted box highlights the TFRs with statistically significant differences, corresponding to the *t* value distribution map. Violin plots overlaid on box plots comparing the normalized RPC between controls and epilepsy patients. Normalized RPC values were extracted for each subject within the time-windows and frequency bands that showed statistically significant differences across all participants. ***D***, Group-normalized average power for controls and epilepsy averaged from the 10–230 ms poststimulus time-window separately for beta (left panel) and 10- to 220 ms poststimulus time-window beta band (middle panel). Group-normalized average power time course for beta and NBG ranges (right panel; line represents groups; shading reflects SEM). Boxplots display the median (horizontal line) and interquartile range (25th and 75th percentiles as box edges), and whiskers extend to the minimum and maximum values, excluding outliers. Outliers are indicated as individual points beyond 1.5 times the interquartile range. The overlaid violin plots show the distribution density of the data for each group. *p*, *p* value; *p < 0.05, Wilcoxon's rank-sum test. SEM, standard error of the mean.

We observed reduced relative power in EPs compared with controls, particularly in beta band and NBG determined from the VIFs (Fig. 5*A*). Specifically, for beta band, the suppression of relative power was observed from ∼50 till ∼330 ms after stimulation onset between ∼15 and ∼21 Hz for the beta band (*p* = 0.009, corrected; Fig. 5*A*). Within this time and frequency window, the group-normalized relative change in power was 0.51 ± 0.16 a.u. in controls versus 0.25 ± 0.09 a.u. in the EP group (*p* = 0.005; Fig. 5*A*). Similarly, for NBG determined from the VIFs, the relative power suppression was observed from ∼10 till ∼200 ms after the stimulation onset between ∼45 and ∼90 Hz (*p* = 0.019, corrected; Fig. 5*A*). Normalized relative changes in power values within this time and frequency window was 0.23 ± 0.17 a.u. in controls versus 0.14 ± 0.11 a.u. in the EP group (*p* = 0.0004; Fig. 5*A*). To accurately assess power changes poststimulation between the two groups, we extracted and averaged power values within time-windows that showed significant differences. Specifically, NBG responses were quantified within the 10–200 ms and beta band responses within the 50–300 ms poststimulus window, where group differences were evident (Fig. 5*B*). Additionally, to characterize the temporal evolution of power changes in each group, we averaged power within defined time bins (−200 to 500 ms) and projected the normalized power changes over time. This allowed us to depict how induced responses differ across groups in both frequency- and time-resolved dimensions (Fig. 5*B*).

Similarly, cluster-based permutation analysis of induced TFRs from VIPs revealed reduced relative power in the EP group (compared with controls) in beta band and NBG (Fig. 5*C*). In beta band, relative power suppression occurred between ∼10 and ∼230 ms poststimulus, within the ∼15 till ∼26 Hz range (*p* = 0.037, corrected; Fig. 5*C*), with reduced power changes in the EPs than in controls (EP 0.29 ± 0.11 a.u. vs controls 0.4 ± 0.19 a.u.; *p* = 0.006; Fig. 5*C*). For the NBG, suppression was evident between ∼10 and ∼220 ms poststimulus, within the ∼30 till ∼50 Hz frequency range (*p* = 0.041, corrected; Fig. 5*C*). During this time and frequency window, relative power change after stimulus onset was lower in the EPs than in controls (EP 0.19 ± 0.16 a.u. vs controls 0.26 ± 0.17 a.u.; *p* = 0.0002; Fig. 5*C*). No significant differences in induced BBG TFRs were found between controls and EPs (*p* > 0.05, cluster-based permutation analysis). To assess power changes poststimulation between groups, we extracted and averaged power values within time-windows that showed significant differences. Specifically, NBG and beta band responses were quantified within the 10–230 ms poststimulus window, where group differences were evident (Fig. 5*D*). Additionally, to characterize the temporal evolution of power changes in each group, we averaged power values within defined time bins (−200 to 500 ms) and projected the normalized power changes over time. This allowed us to understand precisely how induced responses differ across groups in both frequency- and time-resolved dimensions (Fig. 5*D*).

### Characteristics of induced responses between EPs and controls

**Figure 6.**
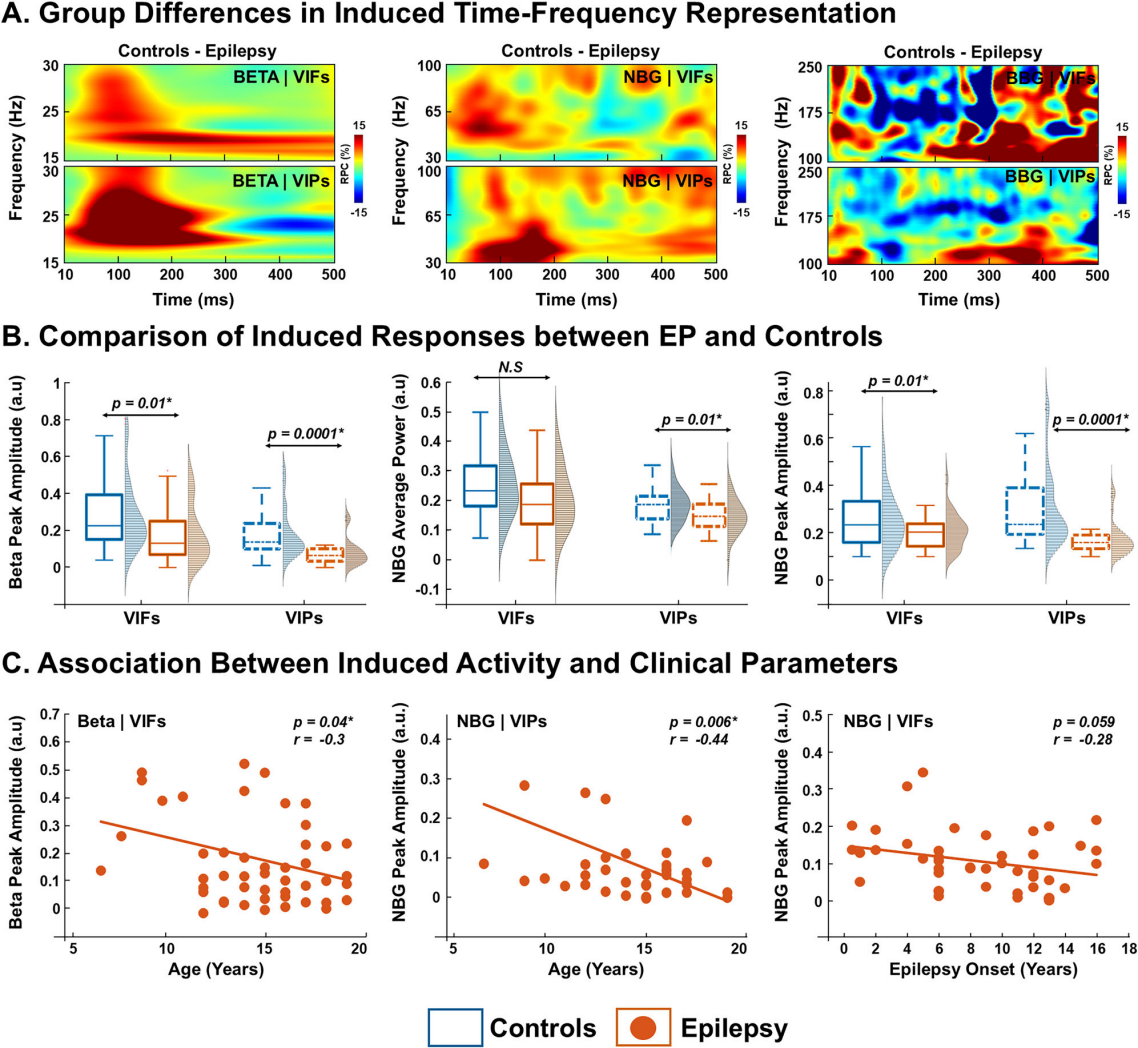
Features of induced beta, narrowband, and broadband oscillations in the healthy controls and epilepsy patients determined by VIFs and potentials. ***A***, The time–frequency plot shows difference in grand-averaged visual responses between neurotypical controls and epilepsy for beta, NBG, and BBG responses determined separately form VIFs (top panel), and VIPs (bottom panel). ***B***, Comparison of induced features (average power and peak amplitude) of beta, NBG, and BBG responses between controls (blue) and epilepsy (orange) determined with MEG (VIFs, solid traces) and EEG (VIPs, dotted traces). ***C***, Scatterplots show the relationship between induced features (determined from VIFs and VIPs) in different frequency ranges (beta, NBG, and BBG) and patient's age and clinical parameter (i.e., age of epilepsy onset), for epilepsy patients (orange dots). In the figure, boxplots display the median (horizontal line) and interquartile range (25th and 75th percentiles as box edges), and whiskers extend to the minimum and maximum values, excluding outliers. Outliers are indicated as individual points beyond 1.5 times the interquartile range. *p*, *p* value; *p < 0.05, Wilcoxon's rank-sum test followed by FDR correction. *r*, Spearman’s correlation coefficient. The color scale represents percentage power change relative to the baseline (−200 to 0 ms).

Induced TFRs computed for VIFs and VIPs used similar Morlet parameters as evoked responses (see Materials and Methods). A cluster-based permutation test revealed prominent power changes in the combined time-window of ∼10–330 ms for beta band and ∼10–220 ms for NBG range, across VIFs and VIPs (Fig. 5*A*,*C*). In this time-window, TFR features for beta band, NBG, and BBG were extracted and compared between EPs and controls (“Wilcoxon rank-sum test”), followed by FDR correction and correlated with age and clinical parameters (“Spearman's,” *p* < 0.05; Fig. 6).

To investigate group differences in induced beta band activity, we first computed the difference in poststimulation responses between groups (Fig. 6*A*). The group-normalized beta peak amplitude was higher in controls compared with EPs for both VIFs and VIPs (VIFs, 0.22 ± 0.23 a.u. vs 0.13 ± 0.17 a.u.; *p* = 0.01; VIPs, 0.13 ± 0.19 a.u. vs 0.06 ± 0.19 a.u.; *p* = 0.0001, FDR-corrected; Fig. 6*B*). No differences in beta average power, peak amplitude, and latency were observed between groups for neither VIFs nor VIPs (*p* > 0.05; Fig. S4*A*). Additionally, in the EP group, beta peak amplitude derived from VIFs showed a moderate negative correlation with age (*r* = −0.30; *p* = 0.04; Fig. 6*C*) and a moderate positive correlation with the ASM number (*r* = 0.37; *p* = 0.008; Fig. S4*B*). Similarly, beta peak frequency was positively correlated with epilepsy duration (VIFs, *r* = 0.33; *p* = 0.025), while beta peak latency was negatively correlated with age of epilepsy onset (*r* = –0.38; *p* = 0.02; Fig. S4*B*).

To assess group differences in induced NBG activity, we subtracted poststimulation responses of the EP group from those of the control group (Fig. 6*A*). Control participants showed a greater increase in NBG power compared with EPs for VIPs only. Specifically, NBG power increased by 0.19 ± 0.1 a.u. in controls versus 0.13 ± 0.16 a.u. in EPs determined from VIPs (*p* = 0.01, FDR-corrected; Fig. 6*B*). Additionally, the peak amplitude of NBG was higher in controls compared with EP for both VIFs (0.24 ± 0.17 a.u. vs 0.2 ± 0.07 a.u.; *p* = 0.01) and VIPs (0.23 ± 0.2 a.u. vs 0.15 ± 0.14 a.u.; *p* = 0.0001, FDR-corrected; Fig. 6*B*). No differences in NBG peak frequency and latency were observed between groups for both VIFs and VIPs (*p* > 0.05, FDR-corrected; Fig. S4*C*). Additionally, in the EP group, NBG peak amplitude derived from VIPs showed a moderate negative correlation with age (*r* = −0.44; *p* = 0.006; Fig. 6*C*). Similarly, NBG peak latency derived from VIPs was moderately and negatively correlated with age of epilepsy onset (*r* = –0.37; *p* = 0.03; Fig. S4*C*).

No differences in BBG average power, peak frequency, amplitude, and latency as determined by VIFs and VIPs were observed between groups (*p* > 0.05; Fig. S4*D*).

### Comparative analysis in focal versus generalized epilepsy

To understand how the features of evoked and induced activity differ between focal and generalized epilepsy relative to controls, we performed subgroup analyses using groupwise “Wilcoxon rank-sum tests” followed by FDR correction (*p* < 0.05; Figs. S3, S4). Furthermore, we assessed whether any clinical parameters and patients' age differed between focal and generalized epilepsy (Table 1). No such difference was observed between focal and generalized epilepsy: (1) age of epilepsy onset (8 ± 4.89 vs 10.2 ± 3.15; *p* = 0.12); (2) patients' age (14.6 ± 3.06 vs 14.5 ± 2.85; *p* = 0. 85); (3) epilepsy duration (7 ± 4.3 vs 5 ± 3.65; *p* = 0. 15); and (4) ASM count (2.5 ± 1.25 vs 2.12 ± 1.08; *p* = 0. 29).

For NBG-evoked responses, group-normalized average power in the VEFs was higher in controls compared with focal EPs (0.04 ± 0.15 vs 0.025 ± 0.06; *p* = 0.004, FDR-corrected; Fig. S3*E*). In contrast, no difference was found between controls and the generalized EPs (0.04 ± 0.15 a.u. vs 0.0258 ± 0.016 a.u.; *p* = 0.07, FDR-corrected; Fig. S3*E*). A similar suppression of evoked peak amplitude was observed between controls and the focal EPs in both VEFs and VEPs, while no differences were found in generalized EPs. Specifically, peak amplitude was reduced in focal EPs compared with controls for VEFs (0.039 ± 0.14 a.u. vs 0.018 ± 0.07 a.u.; *p* = 0.016, FDR-corrected; Fig. S3*E*) and for VEPs (0.06 ± 0.20 a.u. vs 0.01 ± 0.07 a.u.; *p* = 0.019, FDR-corrected; Fig. S3*E*). Additionally, a prolonged latency was observed in the generalized EPs compared with controls for VEFs (83 ± 32 ms vs 109 ± 37 ms; *p* = 0.009, FDR-corrected; Fig. S3*E*), whereas no latency difference was found between controls and the focal EPs. Moreover, no group differences were observed in evoked features across the beta band and BBG for controls, focal, and generalized EPs (*p* > 0.05; Fig. S3*D*,*F*).

Similarly, for beta-induced responses derived from VIFs and VIPs, a suppression of group-normalized peak amplitude was observed in the generalized EPs compared with controls (VIFs, 0.22 ± 0.23 a.u. vs 0.07 ± 0.14 a.u.; *p* = 0.003, FDR-corrected; Fig. S4*E*). Additionally, VIP-derived beta responses revealed a similar suppression of peak amplitude in both focal and generalized EPs compared with controls (controls, 0.13 ± 0.19 a.u.; focal EPs: 0.07 ± 0.10 a.u., *p* = 0.002; generalized EPs, 0.03 ± 0.039 a.u., *p* = 0.02, FDR-corrected; Fig. S4*E*). Likewise, for NBG-induced responses derived from VIFs, a suppression of group-normalized peak amplitude was observed in the generalized EPs compared with controls (VIFs, 0.13 ± 0.18 a.u. vs 0.02 ± 0.09 a.u.; *p* = 0.02; FDR-corrected; Fig. S4*F*). Similarly, NBG peak amplitude derived from VIPs was reduced in both focal and generalized EPs compared with controls (VIPs, controls 0.13 ± 0.20 a.u. vs focal EP 0.06 ± 0.15 a.u.; *p* = 0.039; generalized EP, 0.04 ± 0.10 a.u.; *p* = 0.0004, FDR-corrected; Fig. S4*F*). Moreover, no group differences were observed in induced features across BBG for controls, focal, and generalized epilepsy (*p* > 0.05; Fig. S4*G*).

Furthermore, we used multiple linear regression to assess the influence of clinical variables on normalized evoked and induced features. Patient age, age at epilepsy onset, epilepsy duration, number of ASMs, and epilepsy subtype were included as predictors. No significant associations were observed (*p* < 0.05).

### Comparative oscillatory dynamics in nonlesional epilepsy and controls

To understand how the features of evoked and induced activity differ between nonlesional EPs and controls, we performed subgroup analyses using groupwise “Wilcoxon rank-sum tests” followed by FDR correction (*p* < 0.05; Fig. S5). This comparison focused on patients with uncharacterized epilepsy pathology (*n* = 25; Table 1) to minimize structural confounds and enable clearer assessment of functional disruptions compared with controls during visual stimulation, particularly in beta, NBG, and BBG activity.

Evoked beta band response derived from VEPs showed higher average power in controls compared with the nonlesional EPs (VEPs, 0.25 ± 0.23 a.u vs 0.09 ± 0.19 a.u; *p* = 0.02, FDR-corrected; Fig. S5*A*). Similarly, group-normalized peak amplitude was also higher for controls compared with the nonlesional EPs as determined from VEPs (VEPs, 0.2 a.u. ± 0.18 vs 0.11 ± 0.24 a.u.; *p* = 0.02, FDR-corrected; Fig. S5*A*). No differences were observed in beta peak frequency or latency across groups (*p* > 0.05). Controls exhibited greater evoked NBG average power than the nonlesional EP group for both VEFs (0.04 ± 0.15 a.u. vs 0.02 ± 0.06 a.u.; *p* = 0.0002) and VEPs (0.08 ± 0.23 a.u. vs 0.05 ± 0.14 a.u.; *p* = 0.002, FDR-corrected; Fig. S5*B*). Similarly, group-normalized evoked NBG peak amplitude was higher in controls compared with the nonlesional EPs for both VEFs (0.04 ± 0.14 a.u. vs 0.01 ± 0.07 a.u.; *p* = 0.001) and VEPs (0.06 ± 0.20 a.u. vs 0.02 ± 0.08 a.u.; *p* = 0.002; Fig. S5*B*). Additionally, latency was prolonged in the nonlesional EPs compared with controls, as observed in VEFs (106 ± 45 ms vs 83 ± 32 ms; *p* = 0.002, FDR-corrected; Fig. S5*B*). No difference in NBG peak frequency was observed between controls compared with the nonlesional EPs as determined from VEFs and VEPs (*p* > 0.05). For evoked BBG responses, group-normalized peak amplitude was higher in controls compared with the nonlesional EPs, but only for VEFs (0.16 ± 0.20 a.u. vs 0.11 ± 0.12 a.u.; *p* = 0.01, FDR-corrected; Fig. S5*C*). No differences were observed in BBG average power, peak frequency, or latency between groups in either VEFs or VEPs (*p* > 0.05; Fig. S5*C*).

Induced beta band response derived from VIPs showed higher average power in controls compared with the nonlesional EPs (0.13 ± 0.19 a.u vs 0.08 ± 0.07 a.u; *p* = 0.01, FDR-corrected; Fig. S5*D*). Similarly, group-normalized peak amplitude was also higher for controls compared with the nonlesional EPs as determined from VIPs (VIPs, 0.14 ± 0.19 a.u. vs 0.06 ± 0.07 a.u.; *p* = 0.0003, FDR-corrected; Fig. S5*D*). For NBG-induced responses, controls exhibited greater induced NBG average power than the nonlesional EPs determined from VIFs only (0.5 ± 0.15 a.u. vs 0.32 ± 0.06 a.u.; *p* = 0.004, FDR-corrected; Fig. S5*E*). Similarly, group-normalized induced NBG peak amplitude was higher in controls compared with the nonlesional EPs for VIPs only (0.13 ± 0.2 a.u. vs 0.05 ± 0.06 a.u.; *p* = 0.00002; Fig. S5*E*). No differences were observed in induced BBG average power, peak frequency, amplitude, or latency between groups in either VIFs or VIPs (*p* > 0.05).

### SVM classifier performance

**Figure 7.**
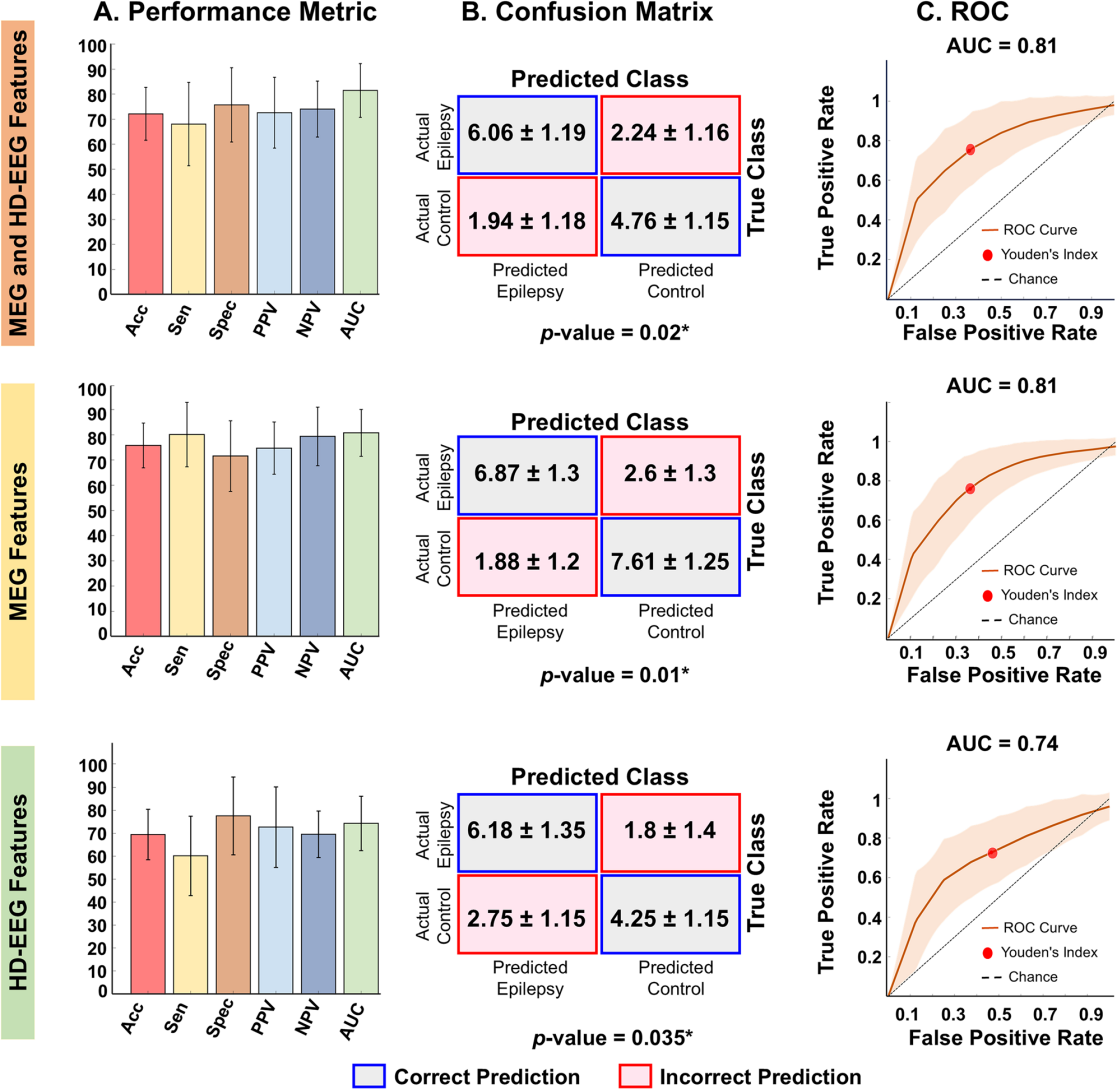
Classification performance of MEG and HD-EEG features for discriminating epilepsy patients versus neurotypical controls. ***A***, Performance metrics (determined from 1,000 iterations), including accuracy (Acc), sensitivity (Sen), specificity (Spec), positive and negative predictive values (PPV and NPV), for combined MEG and HD-EEG features (top panel), MEG features (Middle panel), HD-EEG features (bottom panel). ***B***, Confusion matrices (determined from 1,000 iterations) showing classification results for epilepsy and control groups, with statistical significance (*p* < 0.05, *χ*^2^ test). Results are shown for combined MEG and HD-EEG features (top panel, *n* = 78), MEG features alone (middle panel, *n* = 96), and HD-EEG features alone (bottom panel, *n* = 79), where *n* represents the total number of participants in both groups. ***C***, ROC curves (1,000 iterations; orange traces) with AUC values, indicating classification performance, for combined MEG and HD-EEG features (top panel), MEG features (middle panel), and EEG features (bottom panel). The diagonal dotted line (black traces) represents the chance level; the red circular dot represents Youden’s index.

We evaluated SVM performance across three groups: (1) children with MEG and HD-EEG (Group 1), (2) MEG only (Group 2), and (3) HD-EEG only (Group 3), using ANOVA-selected features with FDR correction (see Materials and Methods). Furthermore, to control for ASM-related bias, we excluded features associated with ASM use (Figs. S3, S4). The number of significant features identified by ANOVA varied across groups: seven in Group 1, six in Group 2, and five in Group 3 (Table S2). Classification accuracy, assessed using 10-fold cross-validation, was highest for Group 2 followed by Group 1 (76 ± 8% for MEG only and 72 ± 10% for MEG combined with HD-EEG) and lowest for Group III (HD-EEG only, 70 ± 11%; Fig. 7*A*). Group II showed a classification performance with a PPV of 75 ± 10% and NPV of 79 ± 11% (*p* < 0.05). For Group 1, PPV was 72 ± 14%, and NPV was 74% ± 11% (*p* < 0.05), while Group 3 had a PPV of 73 ± 17% and NPV of 70 ± 10% (*p* < 0.05; Fig. 7*A*). The ROC curve analysis demonstrated the highest classification model performance for Group 2 (AUC = 0.81 ± 0.9) and Group 1 (AUC = 0.81 ± 0.11) followed by Group 3 (AUC =v0.74 ± 0.12; Fig. 7*C*). To statistically compare model performance, we conducted bootstrap-based AUC comparisons (*n* = 10,000 samples). No significant differences were between the model performance, Group 1 versus Group 2 (*p* = 0.49); Group 1 versus Group 3 (*p* = 0.49); and Group 2 versus Group 3 (*p* = 0.4).

To further assess the added value of MEG or EEG features, we performed a logistic regression analysis within Group 1. The model using EEG-only features had a deviance of 77.13, while the model combining EEG and MEG features showed a reduced deviance of 64.4. A likelihood ratio test comparing these models revealed a statistically significant improvement in model fit (Δdeviance = 12.73; df = 3; *p* = 0.0053), indicating that MEG features added predictive value beyond EEG alone.

## Discussion

By using two independent electrophysiological methods, we showed that brain oscillatory dynamics are altered in EPs compared with neurotypicals. These differences are evident in the power, amplitude, and latency of evoked and induced oscillatory responses in beta and gamma frequency bands. Utilizing group-level differences among different epilepsy subtypes, we trained an SVM classifier to differentiate EPs from controls with high accuracy.

### Temporal and amplitude variations in evoked responses

We observed distinct neurophysiological alterations in EPs during visual stimulation, including reduced amplitudes in late “M250” visual responses and prolonged latency in early and intermediate cortical responses. These differences, identified through VEFs and VEPs, may reflect impaired visual processing (Takeguchi et al., 2024) and align with prior reports of reduced amplitude and latency of early somatosensory responses due to impaired inhibition (Gaetz et al., 2017). The decreased amplitudes of “M250” likely indicate dysfunction in inhibitory interneurons, essential for enhancing neuronal signals during sensory processing (Wood et al., 2017). Inhibitory hypofunction in epilepsy affects cortical signal amplification, leading to weakened visual-evoked responses (Panda and Sharawat, 2020). The “M150” response is linked to feedback processing from V_1_ to dorsal visual streams (Pennartz et al., 2023), which is preferentially detected by MEG due to its sensitivity to tangential currents (Ahlfors et al., 2010). In EPs, the latency differences of “M100” and “M150” components observed in VEFs, along with the delay in the “N1” peak (analogous to “M100”), highlight disruptions in early visual processing. Yet, the more consistent latency delays observed in VEFs likely reflect MEG’s preferential sensitivity to tangential sources, enabling the detection of subtle timing abnormalities that may be attenuated (or spatially smeared) in EEG (Chowdhury et al., 2015). These findings are parallel to somatosensory P50m deficits reported in epilepsy and underscore the MEG’s utility in mapping abnormal functional plasticity (Gaetz et al., 2017). These findings support a dual-pathology model: (1) reduced amplitudes due to synaptic inhibition or adaptation to cortical hyperexcitability and (2) delayed latencies reflecting network dysregulation. Prolonged “M100,” “M150,” and “N1” latencies may serve as noninvasive biomarkers for assessing disrupted feedback integration in epilepsy. This interpretation is supported by diffusion MRI studies showing that reduced auditory tract fractional anisotropy correlates with delayed “M100” latency (Roberts et al., 2010). However, direct correlations between diffusion MRI measures and visual-response latencies have yet to be established.

### Suppression of evoked responses in epilepsy

Evoked response arises from the interplay between a stimulus-driven components and spontaneous brain activity, reflecting sensory processing and intrinsic neural dynamics (Ferezou and Deneux, 2017). Our study shows suppressed visual-evoked activity (i.e., lower peak amplitude and prolonged latency) in pediatric epilepsy in the gamma bands. Our findings suggest widespread disruption in generating stimulus-locked gamma oscillations, potentially reflecting impaired synchronization across visual networks (Kupers et al., 2021).

Gamma oscillations are essential for cortical network coordination (Bitzenhofer, 2023); in epilepsy, disrupted E/I balance may weaken these responses through disinhibition or impaired neural synchronization (Foss-Feig et al., 2017). Here, suppressed evoked activity observed in EPs closely resembles auditory-evoked gamma power suppression seen in Dravet syndrome, a neurodevelopmental disorder often associated with E/I imbalance (Sanchez-Carpintero et al., 2020). Furthermore, subgroup analysis showed distinct patterns of gamma band disruptions: focal epilepsy showed reduced NBG power and peak amplitude, suggesting localized cortical dysfunction, while generalized epilepsy showed prolonged NBG peak latency, indicating delayed network coordination rather than outright suppression, consistent with previous studies (Panda and Sharawat, 2020). Nonlesional epilepsy also exhibited reduced NBG power and amplitude, possibly because of a disrupted functional network (Corona et al., 2023; Rijal et al., 2023). These findings highlight NBG-evoked metrics as noninvasive indicators of early sensory disruption across epilepsy subtypes and etiologies.

We also observed modality-specific differences in the sensitivity of evoked oscillatory dynamics. Group-level VEF differences were most pronounced in both NBG and BBG, whereas VEPs demonstrated differences in beta and NBG bands. These modality-specific patterns likely reflect MEG’s sensitivity to tangential, high-frequency signals, while EEG is more sensitive to radial sources and offers broader cortical coverage (Hauk et al., 2022). The complementary VEF/VEP patterns highlight the benefit of simultaneous recordings in capturing epileptic network dysfunction.

### Suppressed power and amplitude in induced responses

Induced activity provides insights into large-scale network interactions through changes in oscillatory power (David et al., 2006). In our study, EPs showed distinct disruptions in induced responses localized to V_1_, with reduced beta and NBG power and amplitudes, consistent with previous findings of beta power reductions in auditory cortex areas after auditory stimulation in epilepsy cohorts (Findlay et al., 2012).

Epilepsy subtype-specific analysis showed induced beta suppression was most pronounced in generalized epilepsy, reflecting thalamocortical dysrhythmia with widespread cortical disruptions (Lindquist et al., 2023). These abnormalities were present even in nonlesional cases, highlighting their sensitivity to functional disturbance independent of structural damage. In benign epilepsy, reduced beta power extended beyond the seizure onset zone to other brain regions (Song et al., 2019), suggesting broader cortical dysfunction. We also found reduced beta amplitude and power in V_1_, possibly reflecting disrupted connectivity with higher-order regions involved in sensory integration (Cardon, 2018).

NBG oscillations emerge from local interactions between excitatory pyramidal neurons and inhibitory interneurons via rhythmic feedback or interneuron synchronization (van van Hugte et al., 2023). Children with epilepsy showed reduced NBG power and amplitude poststimulus, indicating the initial gamma burst elicited by visual input is suppressed or delayed. These reductions were observed across focal, generalized, and nonlesional epilepsy, likely reflecting disrupted interneuron-mediated synchrony that impairs early sensory processing (Killory et al., 2011).

### Association with clinical parameters

ASMs modulate cortical oscillations by targeting excitatory glutamatergic transmission, enhancing inhibitory networks, or suppressing neuronal excitability through GABAergic inhibition (Löscher and Klein, 2021). In our study, the higher number of ASMs was associated with lower evoked beta frequency and increased induced beta amplitude, suggesting these features may be influenced by ASMs. This indicates beta-evoked and induced responses are more sensitive to ASMs than gamma responses, consistent with evidence that ASMs modulate specific frequencies of cortical excitability and sensory processing (Lanzone et al., 2020). Notably, no group-level association with ASMs was observed considering other clinical parameters.

Our findings underscore the critical role of age in epilepsy-related oscillatory dynamics, with older children showing reduced induced beta and NBG amplitudes. This aligns with previous work showing that gamma and beta oscillations decline with advancing age due to synaptic pruning and structural maturation (Dawood, 2025). Longer epilepsy duration was associated with reduced evoked beta power and BBG amplitude, along with prolonged NBG latency and increased induced beta frequency, suggesting progressive network reorganization. In contrast, later age of epilepsy onset was associated with increased evoked beta and NBG amplitudes and reduced latency, indicating that cortical dysfunction in pediatric epilepsy evolves with brain maturation and may present more dynamic abnormalities that adult-onset epilepsy (Fritschy, 2008).

The proposed biomarkers may aid in localizing epileptogenic zone in complex, MRI-negative cases of drug-resistant epilepsy. Our MRI-negative patients demonstrated reduced beta and NBG power and amplitude of evoked and induced responses, with modality-specific findings in MEG and EEG. These results indicate that functional oscillatory abnormalities can occur without visible lesions on MRI. Thus, MEG and EEG may support individualized treatment planning, including surgery or neuromodulation strategies. Yet, validation with invasive EEG and postsurgical outcome is necessary to confirm their clinical utility and accuracy.

### Distinguish EPs from controls

Prior noninvasive studies achieved moderate accuracy in distinguishing epilepsy from neurotypicals during resting-state conditions (Khalid et al. 2016 ; Shakeshaft et al., 2022). Here, we used features of evoked and induced responses from combined HD-EEG and MEG (Group 1), MEG only (Group 2), and HD-EEG only (Group 3). No AUCs differences were observed across group models. However, logistic regression within Group 1 showed that MEG features improved classification performance compared with EEG features alone, indicating added discriminative value from MEG. Cortical gamma oscillations are low in amplitude and highly localized, making them challenging to capture with EEG (Muthukumaraswamy and Singh, 2013). In contrast, MEG offers a superior signal-to-noise ratio and spatial resolution for detecting superficial, high-frequency cortical sources (Chowdhury et al., 2015). While EEG alone performance was lower, EEG inclusion still contributed valuable information, particularly in the beta and NBG range for evoked and induced responses, where consistent group-level differences were observed. However, generalizability of our findings is limited without external validation, highlighting the need for replication in independent datasets across acquisition sites or conditions.

### Limitations

We did not include neurochemical imaging, limiting assessment of direct links between GABAergic function and oscillatory dynamics. Future multimodal studies are necessary to determine whether the E/I relationship with cortical oscillations is developmentally preserved or altered in epilepsy. Additionally, several patients in our cohort were receiving ASMs, potentially confounding neurophysiological and metabolic measures. The inclusion of a drug-naive cohort would have isolated epilepsy-specific alterations, yet this was not feasible due to the clinical necessity of ongoing treatment. Moreover, classification by ASMs mechanism was not possible, as most patients received individualized ASMs targeting diverse pathways. To address this limitation, we accounted for the ASM number prescribed to each patient as a proxy for overall ASMs. Despite ongoing treatment, evoked and induced oscillations remained suppressed in EPs, suggesting these changes reflect core epilepsy-related pathophysiology rather than medication-related biases.

### Conclusion

By utilizing two electrophysiological modalities, we demonstrate, for the first time, that brain oscillatory dynamics are altered in EPs compared with controls. We interpret differences in evoked and induced responses as functional proxies potentially reflecting disrupted E/I dynamics. Our findings support the potential use of noninvasively measured cortical oscillations as indirect functional proxies of underlying E/I dysfunction. Such biomarkers may help facilitate early epilepsy diagnosis and monitoring of disease progression.

## Data Availability

Data supporting the results of this study are available from the corresponding author upon request.
